# The role of Qishen Yiqi dripping pills in treating chronic heart failure: An overview of systematic reviews and meta-analyses

**DOI:** 10.3389/fcvm.2022.1001072

**Published:** 2022-10-24

**Authors:** Wensheng Chen, Jiezhen Chen, Yuanping Wang, Jiaqi Yan, Xia Yan, Dawei Wang, Yuntao Liu

**Affiliations:** ^1^Guangdong Provincial Hospital of Chinese Medicine, The Second Affiliated Hospital, Guangzhou University of Chinese Medicine, Guangzhou, China; ^2^Shunde Hospital of Guangzhou University of Chinese Medicine, Guangzhou University of Chinese Medicine, Guangzhou, China; ^3^The First Clinical Medical College of Guangzhou, University of Chinese Medicine, Guangzhou, China

**Keywords:** Qishen Yiqi drop pill, chronic heart failure, systematic review, meta-analyses, overview

## Abstract

**Objectives:**

Evidence from systematic reviews/meta-analyses about the efficacy and safety of Qishen Yiqi (QSYQ) dripping pills in chronic heart failure (CHF) remains unclear. This study comprehensively reviewed available systematic reviews on latest evidence to provide reliable information for the clinical use of QSYQ in CHF.

**Methods:**

The systematic review was performed on studies retrieved from six major medical databases. Eligible studies were evaluated in terms of methodological quality and quality of evidence using the Assessment of Multiple Systematic review 2 (AMSTAR-2) tool, the Risk of Bias in Systematic Reviews (ROBIS) was used to assess the risk of bias, and the Preferred Reporting Items for Systematic Review and Meta-analyses (PRISMA) 2020 was utilized for assessing reporting quality. In addition, the Grading of Recommendations Assessment, Development, and Evaluation (GRADE) was used to determine primary outcome indicators’ evidence quality.

**Results:**

A total of 14 systematic reviews were included in this study, based on which it could be concluded that QSYQ combined with conventional medicine (CM) treatment tended to be superior to CM treatment alone in terms of improving cardiac function-related indices (e.g., increasing the left ventricular ejection fraction [LVEF] and reducing the left ventricular end-diastolic dimension [LVEDD] and left ventricular end-systolic internal diameter [LVESD]), improving the total effective rate and 6-min walking distance (6MWD), and reducing N-terminal pro-brain natriuretic peptide (NT-proBNP). Overall, no serious QSYQ-related adverse events were observed. However, the GRADE results showed “very low” to “moderate” evidence for these outcomes, with no high-quality evidence supporting them. Unsatisfactory results were obtained in terms of methodological quality, risk of bias and reporting quality after assessment using the AMSTAR-2, ROBIS, and PRISMA 2020, limited mainly by deficiencies in the following areas: registration of study protocols, explanation of the inclusion of randomized controlled trials (RCTs), complete and detailed search strategy, list of excluded literature, description of funding sources for inclusion in RCTs, investigation of the impact of risk of bias on the results of meta-analysis, and reporting of potential conflicts of interest.

**Conclusion:**

The efficacy and safety of QSYQ adjuvant therapy in CHF remain to be further clarified due to the lack of high-quality evidence provided by current systematic reviews.

## Introduction

Chronic heart failure (CHF), a common cardiovascular disease, is a severe manifestation or the end stage of various cardiac conditions (1). In developed countries, approximately 1–2% of people over 80 years old suffer from CHF, which has a 1- and 5-year mortality rate of 20.2 and 56.2%, respectively (2, 3). CHF also negatively impacts patients’ quality of life and productivity and increases health care costs and socioeconomic burdens (4). Studies have shown that health care spending on CHF in the US was approximately $31 billion in 2012 and that the total cost for heart failure treatment would rise by 127% between 2012 and 2030 (5). The main drugs currently used to manage CHF are beta-blockers, diuretics, calcium channel blockers, angiotensin-converting enzyme inhibitors (ACE-Is), and angiotensin receptor antagonists. Patients with CHF may also benefit from emerging drugs such as SGLT-2 inhibitors (6) and sacubitril/valsartan (7). These new drugs were shown to improve the prognosis of heart failure and lower all-cause mortality (8). Despite advances in CHF treatment, CHF remains a leading cause of death or disability (9), patients’ mortality and rehospitalization rates remain high, and there is an urgent need to develop new therapeutic agents and treatment strategies.

Qishen Yiqi (QSYQ) dripping pill, a well-known Chinese proprietary medicine, was approved by the China Food and Drug Administration (CFDA) in 2003 (Approval number of CFDA: Z20030139) for treating cardiovascular diseases, especially heart failure (10). Its composition is specified in Table 1, of which astragaloside IV, danshensu and salvianolic acids are the major active ingredients (11). A growing body of systematic reviews (10, 12) of randomized controlled trials (RCTs) has investigated the effectiveness of QSYQ in CHF. Although systematic reviews and meta-analyses are widely regarded as the highest level of evidence in the field of evidence-based medicine, the evidence is based on the design and methodology used for assessing the study endpoints (13). While the value of any systematic review depends largely on the number, quality and heterogeneity of the included studies, systematic reviews with serious flaws in methodological quality might mislead decision-makers (14). Although several systematic reviews on QSYQ for CHF have been published, the methodological quality and strength of evidence of these meta-analyses have not yet been adequately assessed. Therefore, the purpose of this present study was to objectively and comprehensively assess current systematic reviews to determine the efficacy and safety of QSYQ in the treatment of CHF.

**TABLE 1:** Components of QSYQ.

Scientific name	Chinese Pinyin	Chinese name	Latin scientific name	Part and form used	Composition ratio
*Astragalus mongholicus Bunge [Fabaceae]*	Huang Qi	黄芪	*Astragalus membranaceus (Fisch.) Bunge*	Dry root	10
*Salvia miltiorrhiza Bunge [Lamiaceae]*	Dan Shen	丹参	*Salvia miltiorrhiza Bge.*	Dried roots and rhizomes	5
*Panax notoginseng (Burkill) F. H. Chen [Araliaceae]*	San Qi	三七	*Panax notoginseng (Burkill) F. H. Chen ex C. H.*	Dried roots and rhizomes	1
*Dalbergia odorifera T. C. Chen [Fabaceae]*	Jiang Xiang	降香	*Dalbergia odorifera T. Chen*	Dried heartwood of trunk and roots	0.067

## Methods

The protocol for this overview was registered on the website of Open Science Framework (OSF^1^) with a registration number of DOI: 10.17605/OSF.IO/JKGP7. Ethical approval was not required because this was a study on systematic reviews.

### Inclusion criteria for reviews selection

#### Types of studies

All peer-reviewed systematic reviews of RCTs evaluating the efficacy and safety of QSYQ for CHF, published in Chinese or English, were included in this study.

#### Types of participants

Participants aged over 18 and diagnosed with CHF according to existing diagnostic criteria (15–17), with no restrictions on race or sex.

#### Types of interventions

The basic treatment for patients with CHF in the control and intervention groups was based on the recommended conventional medicine (CM) from relevant guidelines (9), such as ACE-Is, angiotensin II receptor antagonists, beta-blockers, diuretics, and calcium channel blockers. The intervention group was treated with QSYQ in combination with the basic treatment, with no restriction on dose, frequency of treatment, or duration.

#### Types of outcome measures

The primary outcomes were left ventricular ejection fraction (LVEF) and total effective rate. The latter was defined as the percentage of patients whose signs and symptoms improved during the treatment period and improvement in the New York Heart Association classification by more than one grade according to the guiding principles for clinical research of new drugs in traditional Chinese medicine (18).

The secondary outcomes were adverse events, left ventricular end-diastolic dimension (LVEDD), left ventricular end-systolic internal diameter (LVESD), 6-min walk distance (6MWD), B-type brain natriuretic peptide (BNP), and N-terminal pro-brain natriuretic peptide (NT-proBNP).

### Exclusion criteria

Systematic reviews with the following criteria were excluded: (1) non-peer-reviewed systematic reviews; (2) QSYQ combined with other herbal medicines in the intervention group; (3) duplicate published studies; (4) protocol studies; and (5) those with unretrievable full text even after contacting the authors.

### Search strategy

Two reviewers conducted a comprehensive search of three Chinese databases (China National Knowledge Infrastructure [CNKI], Wanfang and VIP) and three English databases (PubMed, Embase and Cochrane Library) from their inception to May 3, 2022. The detailed search strategy for each database is shown in Supplementary material 1.

### Study selection and data extraction

After removing duplicates, the two authors independently screened the titles and abstracts and evaluated the full text for potentially eligible studies. For studies with insufficient information, the authors of systematic reviews were contacted. Disagreements were resolved through discussion between the two authors. Relevant data were extracted from each eligible review using a standardized form developed by the team, which included first author and year of publication (country), number of trials (subjects), trial quality assessment methods, interventions, main results, and conclusions. For studies with errors or missing data during data extraction, the authors were contacted by email, and if no response was received, this was indicated in the discussion section. The data extraction was independently performed and cross-checked by the two reviewers, and disagreements were resolved by mutual discussion or discussion with a third evaluator.

### Assessment of methodological quality

The methodological quality of the included literature was assessed according to the Assessment of Multiple Systematic Review 2 (AMSTAR-2) tool (19), which consists of 16 items, of which items 2, 4, 7, 9, 11, 13, and 15 are critical. The entries were described as “yes,” “partially yes” and “no,” and the literature was classified as “high,” “moderate,” “low” or “critically low” according to the compliance status of the entries. (1) No or one noncritical weakness was assessed as “high” quality; (2) more than one noncritical weakness was assessed as “moderate” quality; (3) one critical weakness with or without noncritical weaknesses was assessed as “low” quality and; (4) more than one critical weakness with or without noncritical weaknesses was assessed as “critically low” quality. The two reviewers independently evaluated the systematic reviews and discussed their quality. A third reviewer was queried when necessary.

### Assessment of risk of bias

The Risk of Bias in Systematic Reviews (ROBIS) (20) tool is a new tool for assessing the risk of bias in systematic reviews. The tool is divided into three Phases and consists of 24 entries that assist in determining the risk of bias in the review process, results, and conclusions. Responses to landmark questions are indicated by “yes,” “probably yes,” “could be,” “no” and “no information.” The final determination of the risk of bias was classified as “low,” “high” or “uncertain. The risk of bias was considered “low” if all the landmark questions were answered by “maybe” or “could be.” The risk of bias was considered “high” if the answer to any of the landmark questions was “may or may not” or “no,” and “uncertain” if the information provided was insufficient to make a judgment.

### Assessment of the reporting quality

The Preferred Reporting Items for Systematic Review and Meta-analyses (PRISMA) 2020 statement (21) consists of 27 items, including seven areas of specification such as Title, Abstract, Introduction, Methods, Results, Discussion, and Funding. Each item was assessed as “yes,” “no,” and “partially yes” based on the completion of the systematic review.

### Assessment of quality of evidence

The Grades of Recommendation, Assessment, Development and Evaluation (GRADE) (22) was used to evaluate the quality of evidence for outcome indicators through five downgrading factors: study limitations, imprecision, inconsistency, non-directivity, and publication bias. After the assessment, evidence was categorized into four levels: “high,” “moderate,” “low,” and “very low.” To reach an objective result, evaluators were trained to reach a consensus before conducting the evaluation. The entire evaluation process was conducted by two independent reviewers, and any disagreements were resolved through consensus or discussion with an experienced and authoritative third reviewer.

## Results

### Study selection

**FIGURE 1:**
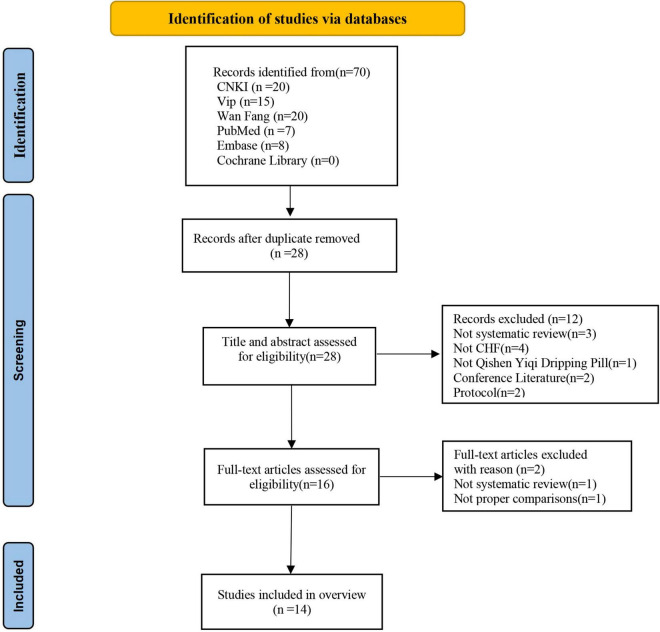
Flow chart of the study selection process. CHF, chronic heart failure; CNKI, China National Knowledge Infrastructure database; Embase, Excerpta Medica database; SR, systematic review.

In total, 70 studies were searched from English and Chinese databases according to predefined search strategies. Twenty-eight studies remained after manually screening out duplicates. After reading the titles and abstracts, 12 studies were excluded as they did not meet the inclusion criteria, of which two studies were conference papers, one study was a research protocol, three studies were not systematic reviews, one study did not use QSYQ, and two studies were not on CHF. Then, the full text of 16 potential articles was further screened, of which one of our previous studies was excluded because it was an RCT. Finally, 14 systematic reviews (10, 12, 23–34) were included. The specific screening process is presented in Figure 1 and Supplementary material 2.

### Characteristics of the included reviews

Table 2 summarizes the main characteristics of all included reviews. They were published between 2013 and 2021, with two studies (10, 12) published in English and the remaining (23–34) published in Chinese. All articles were published in peer-reviewed journals. Of the 14 systematic reviews, 13 (10, 12, 23–31, 33, 34) compared QSYQ in combination with CM to CM alone, and one systematic review (32) compared the efficacy of QSYQ in combination with trimetazidine to trimetazidine alone in addition to CM treatment. Eight studies (10, 23, 25, 30–34) assessed the methodological quality of systematic reviews using the Cochrane Collaboration’s risk of bias tool, and six studies (12, 24, 26–29) used the Jadad scale. Eleven studies (10, 12, 23–26, 29, 30, 32–34) reported the funding source, all of which were government-funded. The results of all systematic reviews suggested that QSYQ appeared to have beneficial effects in CHF treatment but required further support from high-quality RCTs.

**TABLE 2:** Main characteristics of included studies.

References	Country	Trials (subjects)	Treatment intervention	Control intervention	Quality assessment	Meta analysis	Language of publication	Funding	Main results
Qiuyue et al. (28)	China	8 (788)	QSYQ + CM	CM	Jadad	Yes	Chinese	NR	QSYQ can improve LVESD and LVEDD in patients with CHF.
Chen et al. (10)	China	21 (2162)	QSYQ + CM	CM	Cochrane criteria	Yes	English	Yes	QSYQ combined with CM are better than conventional medicine alone to improve the indicators of patients with CHF.
Genhao et al. (29)	China	17 (1701)	QSYQ + CM	CM	Jadad	Yes	Chinese	Yes	QSYQ has been shown to be effective and safe in the treatment of heart failure patients with coronary artery disease in combination with CM.
Zhangchun et al. (26)	China	13 (1541)	QSYQ + CM	CM	Jadad	Yes	Chinese	Yes	QSYQ is safe and effective in the treatment of congestive heart failure.
Jiao et al. (30)	China	27 (2726)	QSYQ + CM	CM	Cochrane criteria	Yes	Chinese	Yes	QSYQ is clinically effective in the treatment of CHF with no adverse effects.
Xuejing et al. (31)	China	27 (3893)	QSYQ + CM	CM	Cochrane criteria	Yes	Chinese	Yes	QSYQ significantly increased the efficiency, LVEF and 6WMD in patients with CHF compared to CM.
Jungang et al. (25)	China	18 (2244)	QSYQ + CM	CM	Cochrane criteria	Yes	Chinese	NR	The addition of QSYQ to CM therapy improves cardiac function, increases CO, reduces LVEDD and LVESD, and reduces readmission rates in CHF patients.
Zhenchao et al. (34)	China	12 (1946)	QSYQ + CM	CM	Cochrane criteria	Yes	Chinese	Yes	QSYQ in combination with CM was more effective in improving patients’ NYHA cardiac outcomes, left ventricular ejection fraction, NT-proBNP and 6-MWD compared to CM.
Yinghao et al. (24)	China	8 (948)	QSYQ + CM	CM	Jadad	Yes	Chinese	Yes	QSYQ plus CM versus CM alone for CHF may further improve clinical outcomes, but the quality of evidence is low and evidence from high quality studies is still needed to support this.
Ye and Jianxia (27)	China	15 (1614)	QSYQ + CM	CM	Jadad	Yes	Chinese	NR	QSYQ in combination with CM is more effective and safer than CM alone in improving the indicators of heart failure in patients with coronary heart disease.
Wang et al. (12)	China	85 (8579)	QSYQ + CM	CM	Jadad	Yes	English	Yes	QSYQ combined with CM might be effective in CHF patients.
Shuanhu et al. (23)	China	17 (1840)	QSYQ + CM	CM	Cochrane criteria	Yes	Chinese	Yes	The addition of QSYQ to CM may further improve clinical efficacy and safety compared to CM alone in the treatment of CHF.
Feng et al. (32)	China	9 (815)	QSYQ + trimetazidine + CM	Trimetazidine + CM	Cochrane criteria	Yes	Chinese	Yes	QSYQ in combination with trimetazidine was better than trimetazidine alone in improving various parameters in patients with chronic heart failure.
Xiuwen et al. (33)	China	11 (931)	QSYQ + CM	CM	Cochrane criteria	Yes	Chinese	Yes	QSYQ in combination with CM is safe and effective in the treatment of CHF.

QSYQ, Qishen Yiqi dripping Pill; CM, conventional medicine; CHF, chronic heart failure; NR, no report.

### Methodological quality of the included systematic review

Table 3 shows the results of the AMSTAR-2 evaluation of the methodological quality of the 14 systematic reviews included in this overview. As all studies had more than one key weakness, their methodological quality was determined to be critically low. The methodological quality of the systematic reviews varied considerably, with most of the included studies showing certain limitations. Such as, one study (12) did not report clear inclusion and exclusion criteria, none of the studies had registered study protocols on relevant websites prior to conducting the systematic evaluation, none of the studies explained the rationale for including only RCTs in the systematic reviews, and none of the authors of the systematic reviews used a comprehensive search strategy such as searching gray literature. One studies (30) did not use two-person repeated literature screening, seven studies (25, 26, 28–32) did not use two-person repeated extraction of data, no studies provided full exclusion descriptions for excluding the retrieved literature, one study (28) did not sufficiently detail the essential characteristics of the included RCTs and no studies reported on the source of funding for the included RCTs. When conducting the meta-analysis, 12 studies (10, 23–26, 28–34) did not analyze the impact of RCTs with a high risk of bias on the meta-analysis outcomes, and five studies (24, 25, 28, 29, 31) did not consider the effect of the risk of bias in the inclusion of the original RCT when interpreting and discussing the results of the meta-analysis. Five studies (24, 25, 28, 30, 31) did not provide reasonable explanations or discussions on the heterogeneity of the findings, three studies (23, 24, 31) did not perform analysis or provide reasonable discussions on publication bias, and eleven studies (23–33) did not report on all potential sources of conflicts of interest.

**TABLE 3:** Methodological quality assessment of the included reviews using the AMSTAR 2 tool.

References	Q1	Q2	Q3	Q4	Q5	Q6	Q7	Q8	Q9	Q10	Q11	Q12	Q13	Q14	Q15	Q16	Overall quality
Qiuyue et al. (28)	Y	N	N	PY	Y	N	N	N	Y	N	Y	N	N	N	Y	N	CL
Chen et al. (10)	Y	N	N	PY	Y	Y	N	Y	Y	N	Y	N	Y	Y	Y	Y	CL
Genhao et al. (29)	Y	N	N	PY	Y	N	N	Y	Y	N	Y	N	N	Y	Y	N	CL
Zhangchun et al. (26)	Y	N	N	PY	Y	N	N	Y	Y	N	Y	N	Y	Y	Y	N	CL
Jiao et al. (30)	Y	N	N	PY	N	N	N	Y	Y	N	Y	N	Y	N	Y	N	CL
Xuejing et al. (31)	Y	N	N	PY	Y	N	N	Y	Y	N	Y	N	N	N	N	N	CL
Jungang et al. (25)	Y	N	N	PY	Y	N	N	Y	Y	N	Y	N	N	N	Y	N	CL
Zhenchao et al. (34)	Y	N	N	PY	Y	Y	N	Y	Y	N	Y	N	Y	Y	Y	Y	CL
Yinghao et al. (24)	Y	N	N	PY	Y	Y	N	Y	Y	N	Y	N	N	N	N	N	CL
Ye and Jianxia (27)	Y	N	N	PY	Y	Y	N	Y	Y	N	Y	Y	Y	Y	Y	N	CL
Wang et al. (12)	N	N	N	PY	Y	Y	N	Y	Y	N	Y	Y	Y	Y	Y	Y	CL
Shuanhu et al. (23)	Y	N	N	PY	Y	Y	N	Y	Y	N	Y	N	Y	Y	N	N	CL
Feng et al. (32)	Y	N	N	PY	Y	N	N	Y	Y	N	Y	N	Y	Y	Y	N	CL
Xiuwen et al. (33)	Y	N	N	PY	Y	Y	N	Y	Y	N	Y	N	Y	Y	Y	N	CL
Number of Y (%)	13(92.9)	0(0)	0(0)	0(0)	13(92.9)	7(50.0)	0(0)	13(92.9)	14(100)	0(0)	14(100)	2(14.3)	9(64.3)	9(64.3)	11(78.6)	3(21.4)	

Q, question; Y, yes; N, no; PY, partial yes; CL, critically low.

Q1: Did the research questions and inclusion criteria for the review include the components of PICO?

Q2: Did the report of the review contain an explicit statement that the review methods were established prior to the conduct of the review and did the report justify any significant deviations from the protocol?

Q3: Did the review authors explain their selection of the study designs for inclusion in the review?

Q4: Did the review authors use a comprehensive literature search strategy?

Q5: Did the review authors perform study selection in duplicate?

Q6: Did the review authors perform data extraction in duplicate?

Q7: Did the review authors provide a list of excluded studies and justify the exclusions?

Q8: Did the review authors describe the included studies in adequate detail?

Q9: Did the review authors use a satisfactory technique for assessing the risk of bias (RoB) in individual studies that were included in the review?

Q10: Did the review authors report on the sources of funding for the studies included in the review?

Q11: If meta-analysis was performed did the review authors use appropriate methods for statistical combination of results?

Q12: If meta-analysis was performed, did the review authors assess the potential impact of RoB in individual studies on the results of the meta-analysis or other evidence synthesis?

Q13: Did the review authors account for RoB in individual studies when interpreting/discussing the results of the review?

Q14: Did the review authors provide a satisfactory explanation for, and discussion of, any heterogeneity observed in the results of the review?

Q15: If they performed Quantitative synthesis did the review authors carry out an adequate investigation of publication bias (small study bias) and discuss its likely impact on the results of the review?

Q16: Did the review authors report any potential sources of conflict of interest, including any funding they received for conducting the review?

### Risk of bias of included systematic review

Risk of bias in systematic reviews assessment results indicated that in Phase 1, where the relevance of the study topic was assessed, all systematic reviews were rated as low risk of bias. In Phase 2, for Domain 1, One study (12) had a high risk of bias due to the lack of clear inclusion criteria, and all other studies had a low risk of bias. In Domains 2, all systematic reviews were at high risk of bias. In Domain 3, four systematic reviews (25, 30–32) had unclear risks, three (26, 28, 29) had a high risk of bias, and only seven (10, 12, 23, 24, 27, 33, 34) had a low risk of bias. In Domains 4, three systematic reviews (12, 27, 34) had unclear risks, 11 (10, 23–26, 28–33) had a high risk of bias. In addition, in Phase 3, all systematic reviews had a high risk of bias. The evaluation details of the included systematic reviews on the ROBIS scale are shown in Table 4 and Supplementary material 3.

**TABLE 4:** Tabular presentation of risk of bias of included systematic reviews.

References	Phase 1	Phase 2				Phase 3
	Intervention reviews	(1) Study eligibility criteria	(2) Identification and selection of studies	(3) Data collection and study appraisal	(4) Synthesis and findings	Risk of bias in the review
Qiuyue et al. (28)	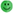	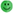	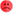	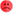	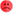	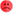
Chen et al. (10)	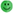	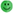	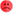	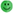	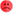	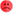
Genhao et al. (29)	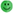	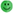	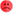	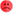	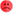	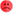
Zhangchun et al. (26)	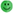	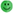	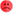	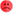	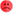	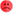
Jiao et al. (30)	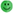	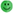	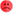	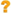	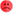	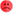
Xuejing et al. (31)	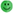	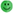	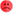	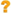	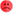	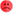
Jungang et al. (25)	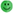	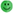	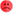	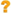	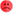	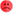
Zhenchao et al. (34)	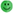	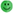	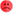	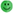	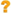	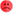
Yinghao et al. (24)	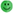	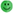	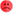	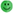	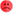	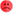
Ye and Jianxia (27)	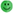	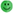	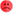	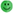	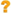	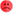
Wang et al. (12)	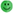	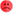	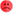	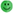	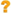	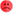
Shuanhu et al. (23)	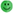	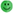	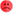	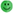	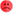	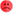
Feng et al. (32)	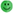	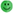	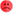	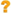	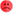	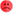
Xiuwen et al. (33)	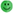	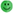	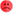	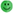	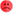	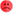

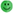
, low risk; 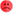, high risk; 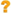, unclear risk.

### Reporting quality of included systematic review

Table 5 presents the results of the quality of the reports assessed by the PRISMA 2020 checklist. Although Q1 (title), Q3 (rationale), Q5 (eligibility criteria), Q13a, b, c d (synthesis of methods), Q16 (study selection), Q17 (study characteristics), Q18 (risk of bias), Q19 (results of individual studies), Q20a, b (results of syntheses) and Q23d (discussion) were fully reported, some reporting deficiencies were found in other sections. Q2 (abstract), Q6 (information sources), Q7 (search strategy), Q15 (certainty assessment), Q22 (certainty of evidence), Q24 (registration and protocol), and Q27 (availability of data, code and other materials) were reported deficiently (0%). The remaining entries are only partially complete.

**TABLE 5:** Results of the PRISMA assessments.

Section/ Topic	Items	Wang et al. (12)	Chen et al. (10)	Zhenchao et al. (34)	Feng et al. (32)	Xiuwen et al. (33)	Jiao et al. (30)	Ye and Jianxia (27)	Genhao et al. (29)	Zhangchun et al. (26)	Qiuyue et al. (28)	Xuejing et al. (31)	Jungang et al. (25)	Yinghao et al. (24)	Shuanhu et al. (23)	Compliance (%)
**Title**																
Title	1	Y	Y	Y	Y	Y	Y	Y	Y	Y	Y	Y	Y	Y	Y	100
**Abstract**																
Abstract	2	PY	PY	PY	PY	PY	PY	PY	PY	PY	PY	PY	PY	PY	PY	0
**Introduction**																
Rationale	3	Y	Y	Y	Y	Y	Y	Y	Y	Y	Y	Y	Y	Y	Y	100
Objectives	4	N	Y	Y	Y	Y	Y	Y	Y	Y	Y	Y	Y	Y	Y	93
**Methods**																
Eligibility criteria	5	Y	Y	Y	Y	Y	Y	Y	Y	Y	Y	Y	Y	Y	Y	100
Information sources	6	N	N	N	N	N	N	N	N	N	N	N	N	N	N	0
Search strategy	7	N	N	N	N	N	N	N	N	N	N	N	N	N	N	0
Selection process	8	Y	Y	Y	N	Y	N	Y	Y	Y	Y	Y	Y	Y	Y	56
Data collection process	9	Y	Y	Y	N	Y	N	Y	N	N	N	Y	N	Y	Y	57
Data items	10a	Y	Y	Y	N	Y	Y	Y	Y	N	Y	Y	Y	Y	Y	86
	10b	Y	Y	Y	N	Y	Y	Y	Y	N	N	Y	Y	Y	Y	79
Study risk of bias assessment	11	Y	Y	Y	Y	Y	Y	Y	N	N	Y	Y	Y	Y	Y	86
Effect measures	12	Y	Y	Y	Y	Y	Y	Y	N	N	Y	Y	Y	Y	Y	86
Synthesis methods	13a	Y	Y	Y	Y	Y	Y	Y	Y	Y	Y	Y	Y	Y	Y	100
	13b	Y	Y	Y	Y	Y	Y	Y	Y	Y	Y	Y	Y	Y	Y	100
	13c	Y	Y	Y	Y	Y	Y	Y	Y	Y	Y	Y	Y	Y	Y	100
	13d	Y	Y	Y	Y	Y	Y	Y	Y	Y	Y	Y	Y	Y	Y	100
	13e	Y	N	Y	N	Y	Y	Y	N	N	N	N	N	N	Y	43
	13f	Y	N	N	N	N	N	Y	N	N	N	Y	N	N	Y	29
Reporting bias assessment	14	Y	Y	Y	N	Y	N	Y	N	Y	N	N	N	N	N	43
Certainty assessment	15	N	N	N	N	N	N	N	N	N	N	N	N	N	N	0
**Results**																
Study selection	16a	Y	Y	Y	Y	Y	Y	Y	Y	Y	Y	Y	Y	Y	Y	100
	16b	Y	Y	Y	Y	Y	Y	Y	Y	Y	Y	Y	Y	Y	Y	100
Study characteristics	17	Y	Y	Y	Y	Y	Y	Y	Y	Y	Y	Y	Y	Y	Y	100
Risk of bias in studies	18	Y	Y	Y	Y	Y	Y	Y	Y	Y	Y	Y	Y	Y	Y	100
Results of individual studies	19	Y	Y	Y	Y	Y	Y	Y	Y	Y	Y	Y	Y	Y	Y	100
Results of syntheses	20a	Y	Y	Y	Y	Y	Y	Y	Y	Y	Y	Y	Y	Y	Y	100
	20b	Y	Y	Y	Y	Y	Y	Y	Y	Y	Y	Y	Y	Y	Y	100
	20c	Y	N	Y	Y	Y	Y	Y	N	N	N	N	N	N	Y	50
	20d	Y	N	Y	N	N	N	Y	N	N	N	Y	N	N	N	29
Reporting biases	21	Y	Y	Y	N	Y	Y	Y	Y	Y	Y	N	N	N	N	64
Certainty of evidence	22	N	N	N	N	N	N	N	N	N	N	N	N	N	N	0
**Discussion**																
Discussion	23a	Y	Y	Y	Y	Y	Y	Y	Y	Y	N	Y	Y	Y	Y	93
	23b	Y	Y	Y	Y	Y	Y	Y	Y	Y	N	Y	Y	Y	Y	93
	23c	Y	Y	Y	Y	Y	N	N	N	Y	N	Y	Y	Y	Y	71
	23d	Y	Y	Y	Y	Y	Y	Y	Y	Y	Y	Y	Y	Y	Y	100
**Other information**																
Registration and protocol	24a	N	N	N	N	N	N	N	N	N	N	N	N	N	N	0
	24b	N	N	N	N	N	N	N	N	N	N	N	N	N	N	0
	24c	N	N	N	N	N	N	N	N	N	N	N	N	N	N	0
Support	25	Y	Y	Y	Y	Y	Y	Y	Y	Y	N	Y	N	Y	Y	86
Competing interests	26	Y	Y	Y	N	N	N	N	N	N	N	N	N	N	N	21
Availability of data, code and other materials	27	N	N	N	N	N	N	N	N	N	N	N	N	N	N	0

Y, yes; N, no; PY, partial yes.

### Quality of evidence in the included systematic reviews

The details of the 14 systematic reviews containing a total of 52 outcomes for GRADE assessment are shown in Table 6. The results showed that 4 (4/52, 7.69%), 43 (43/52, 82.69%), and 5 (5/52, 9.62%) outcomes were rated as moderate, low and very low quality, respectively. No high-quality evidence results were found. Significant risks of bias in the systematic reviews were due to the design of the original RCTs (52/52, 100.00%), which was the most important factor contributing to lower quality of evidence, followed by inconsistency (39/52, 75.00%), publication bias (7/52, 13.46%), and imprecision (5/52, 9.62%).

**TABLE 6:** Quality of evidence in the included systematic reviews based on GRADE.

References	Interventions	Outcomes	Study design	Included RCTs (patients)	Effect estimates 95% CI	*I*^2^(%)	Risk of bias	Inconsistency	Indirectness	Imprecision	Other considerations	Quality of evidence
Shuanhu et al. (23)	QSYQ + CM vs. CM	TER	RCT	7 (767)	RR 1.18 (1.12, 1.25)	0	Serious^a^	Not serious	Not serious	Not serious	None	Model
		6MWD	RCT	7 (882)	WMD 94.39 (71.89, 116.89)	95	Serious^a^	Serious^b^	Not serious	Not serious	None	Low
		BNP	RCT	5 (478)	WMD 194.85 (–52.91, 442.61)	99	Serious^a^	Serious^b^	Not serious	Not serious	None	Low
Chen et al. (10)	QSYQ + CM vs. CM	TER	RCT	20 (2076)	RR 1.21 (1.17, 1.26)	0	Serious^a^	Not serious	Not serious	Not serious	Publication bias strongly suspected^d^	Low
		LVEF	RCT	16 (1590)	MD 6.11 (5.23, 6.99)	69	Serious^a^	Serious^b^	Not serious	Not serious	None	Low
		LVEDD	RCT	11 (1241)	MD –7.48 (–9.71, –5.24)	96	Serious^a^	Serious^b^	Not serious	Not serious	None	Low
		LVESD	RCT	11 (1241)	MD –3.54 (–6.85, –0.24)	98	Serious^a^	Serious^b^	Not serious	Not serious	None	Low
		BNP	RCT	17 (1762)	SMD –2.26 (–2.89, –1.63)	96	Serious^a^	Serious^b^	Not serious	Not serious	None	Low
		6MWD	RCT	7 (638)	MD 106.47 (83.37, 129.57)	94	Serious^a^	Serious^b^	Not serious	Not serious	None	Low
Zhenchao et al. (34)	QSYQ + CM vs. CM	TER	RCT	9 (1361)	RR 1.21 (1.13, 1.30)	0	Serious^a^	Not serious	Not serious	Not serious	Publication bias strongly suspected^d^	Low
		LVEF	RCT	11 (1777)	SMD 0.67 (0.41, 0.93)	84	Serious^a^	Serious^b^	Not serious	Not serious	None	Low
		NT-proBNP	RCT	11 (1328)	SMD –1.45 (–2.00, –1.14)	95	Serious^a^	Serious^b^	Not serious	Not serious	None	Low
		6MWD	RCT	9 (1690)	SMD 1.33 (0.82, 1.85)	95	Serious^a^	Serious^b^	Not serious	Not serious	None	Low
Feng et al. (32)	QSYQ + CM + TMZ vs. CM + TMZ	LVEF	RCT	4 (469)	MD 6.03 (5.39, 6.67)	41	Serious^a^	Serious^b^	Not serious	Not serious	None	Low
		LVESD	RCT	5 (565)	MD –6.62 (–7.11, –6.13)	96	Serious^a^	Serious^b^	Not serious	Not serious	None	Low
		BNP	RCT	2 (295)	MD –101.87 (–109.90, –93.83)	0	Serious^a^	Not serious	Not serious	Serious^c^	None	Low
		6MWD	RCT	2 (125)	MD 110.13 (96.89, 123.36)	0	Serious^a^	Not serious	Not serious	Serious^c^	None	Low
Xiuwen et al. (33)	QSYQ + CM + TMZ vs. CM + TMZ	TER	RCT	4 (373)	RR 1.33 (1.11, 1.58)	68	Serious^a^	Serious^b^	Not serious	Not serious	None	Low
		LVEF	RCT	6 (640)	MD 7.08 (5.87, 8.28)	73	Serious^a^	Serious^b^	Not serious	Not serious	None	Low
		LVEDD	RCT	5 (560)	MD –8.78 (–11.60, –5.96)	96	Serious^a^	Serious^b^	Not serious	Not serious	None	Low
		6MWD	RCT	4 (296)	MD 100.09 (79.40, 120.77)	76	Serious^a^	Serious^b^	Not serious	Serious^c^	None	Very low
Jiao et al. (30)	QSYQ + CM vs. CM	TER	RCT	14 (1441)	OR 4.25 (2.99, 6.04)	0	Serious^a^	Not serious	Not serious	Not serious	None	Model
		LVEF	RCT	22 (2250)	SMD 0.06 (0.04, 0.07)	85	Serious^a^	Serious^b^	Not serious	Not serious	None	Low
		BNP	RCT	12 (1297)	SMD –243.19 (–305.78, –180.59)	100	Serious^a^	Serious^b^	Not serious	Not serious	None	Low
		LVEDD	RCT	10 (1180)	SMD –4.57 (–7.26, –1.88)	96	Serious^a^	Serious^b^	Not serious	Not serious	None	Low
		6MWD	RCT	15 (1467)	SMD 61.3 (35.71, 86.88)	98	Serious^a^	Serious^b^	Not serious	Not serious	None	Low
Ye and Jianxia (27)	QSYQ + CM vs. CM	LVEF	RCT	12 (1275)	MD 6.55 (5.35, 7.74)	62	Serious^a^	Serious^b^	Not serious	Not serious	None	Low
		BNP	RCT	4 (390)	MD –63.55 (–85.48, –41.63)	85	Serious^a^	Serious^b^	Not serious	Serious^c^	None	Low
		6MWD	RCT	11 (1162)	MD 71.37 (53.28, 89.47)	95	Serious^a^	Serious^b^	Not serious	Not serious	None	Low
Genhao et al. (29)	QSYQ + CM vs. CM	LVEF	RCT	9 (851)	MD 6.82 (5.45, 8.19)	79	Serious^a^	Serious^b^	Not serious	Not serious	None	Low
		BNP	RCT	5 (406)	MD –75.9 (–93.14, –58.65)	88	Serious^a^	Serious^b^	Not serious	Not serious	None	Low
		6MWD	RCT	8 (774)	MD 57.86 (29.54, 86.19)	96	Serious^a^	Serious^b^	Not serious	Not serious	None	Low
Zhangchun et al. (26)	QSYQ + CM vs. CM	TER	RCT	13 (1590)	OR 2.75 (2.07, 3.66)	0	Serious^a^	Not serious	Not serious	Not serious	Publication bias strongly suspected^d^	Low
Wang et al. (12)	QSYQ + CM vs. CM	6MWD	RCT	11 (1065)	SMD 2.38 (1.63, 3.13)	96	Serious^a^	Serious^b^	Not serious	Not serious	Publication bias strongly suspected^d^	Very low
		LVEDD	RCT	14 (1665)	SMD –1.34 (–1.87, –0.80)	96	Serious^a^	Serious^b^	Not serious	Not serious	None	Low
		LVESD	RCT	13 (1592)	SMD –0.60 (–1.14, –0.05)	96	Serious^a^	Serious^b^	Not serious	Not serious	None	Low
		LVEF	RCT	24 (2611)	SMD 1.08 (0.84, 1.33)	88	Serious^a^	Serious^b^	Not serious	Not serious	Publication bias strongly suspected^d^	Very low
		BNP	RCT	13 (1464)	SMD –2.90 (–3.76, –2.03)	97	Serious^a^	Serious^b^	Not serious	Not serious	Publication bias strongly suspected^d^	Very low
		NT-proBNP	RCT	6 (645)	SMD –3.58 (–5.15, –2.01)	98	Serious^a^	Serious^b^	Not serious	Not serious	Publication bias strongly suspected^d^	Very low
Qiuyue et al. (28)	QSYQ + CM vs CM	LVESD	RCT	8 (788)	WMD –1.82 (–2.34, –1.30)	7	Serious^a^	Not serious	Not serious	Not serious	None	Model
		LVEDD	RCT	8 (788)	WMD –2.55 (–3.63, –1.47)	71	Serious^a^	Serious^b^	Not serious	Not serious	None	Low
Xuejing et al. (31)	QSYQ + CM vs. CM	TER	RCT	24 (3371)	RR 1.20 (1.16, 1.23)	0	Serious^a^	Not serious	Not serious	Not serious	None	Low
		LVEF	RCT	22 (3266)	MD 6.79 (6.50, 7.07)	91	Serious^a^	Serious^b^	Not serious	Not serious	None	Low
		6MWD	RCT	14 (2197)	MD 45.08 (43.09, 47.08)	99	Serious^a^	Serious^b^	Not serious	Not serious	None	Low
		BNP	RCT	10 (1506)	MD –108.51 (–112.54, –104.49)	99	Serious^a^	Serious^b^	Not serious	Not serious	None	Low
Jungang et al. (25)	QSYQ + CM vs. CM	LVEDD	RCT	6 (510)	SMD –0.54 (–0.76, –0.31)	38	Serious^a^	Serious^b^	Not serious	Not serious	None	Low
		LVESD	RCT	5 (450)	SMD –0.53 (–0.72, –0.34)	0	Serious^a^	Not serious	Not serious	Not serious	None	Low
Yinghao et al. (24)	QSYQ + CM vs. CM	TER	RCT	8 (948)	RR 1.16 (1.10, 1.20)	0	Serious^a^	Not serious	Not serious	Not serious	None	Low
		LVEF	RCT	8 (948)	MD 7.76 (7.47, 8.05)	93	Serious^a^	Serious^b^	Not serious	Not serious	None	Low
		LVEDD	RCT	7 (790)	MD –2,60 (–4.34, –1.76)	76	Serious^a^	Serious^b^	Not serious	Not serious	None	Low
		LVESD	RCT	3 (297)	MD –2.31 (–3.34, –1.27)	0	Serious^a^	Not serious	Not serious	Serious^c^	None	Model
		BNP	RCT	4 (457)	MD –98.49 (–103, –93.9)	27	Serious^a^	Not serious	Not serious	Not serious	None	Model

BNP, brain natriuretic peptide; CM, conventional medicine; LVEDD, left ventricular end-diastolic dimensions; LVEF, left ventricular ejection fraction; LVESD, left ventricular end-systolic dimensions; NT-pro BNP, N-terminal prohormone of BNP; QSYQ, Qishen Yiqi dripping Pill; 6MWD, 6-min walking distance; TMZ, trimetazidine; RR, risk ratio; OR, odds ratio; MD, mean difference; SMD, standardized mean difference; CI, confidence interval; RCT, randomized controlled trial.

*^a^*The design of the experiment with a large bias in random, distributive hiding or blind.

*^b^*The confidence interval overlaps less, the heterogeneity test P is very small, and the *I*^2^ is larger.

*^c^*As OIS criteria were not met, primarily due to the small sample size ( < 400).

*^d^*Asymmetric funnel plot showing publication bias.

### Efficacy evaluation with evidence quality

#### Left ventricular ejection fraction

Ten systematic reviews (10, 12, 24, 27, 29–34) reported on LVEF outcomes, and all meta-analyses showed that combined QSYQ treatment was significantly better than CM alone in improving LVEF in patients with CHF. However, most of the results (9/10, 90%) (10, 12, 24, 27, 29–31, 33, 34) were significantly heterogeneous, which further decreased the strength of the evidence. This outcome in most of the meta-analyses (9/10, 90%) (10, 24, 27, 29–34) was evaluated as a “low” strength of evidence, with one (12) being “very low.” The study with the largest sample size included 22 RCTs (31), with a total of 3,266 patients (MD = 6.79, 95% CI = 6.50 to 7.07, *p* < 0.00001, *I*^2^ = 91%, random effect model). The results were statistically and clinically significant.

#### Total effective rate

Eight systematic reviews (10, 23, 24, 26, 30, 31, 33, 34) compared the total effective rate of QSYQ combined with CM versus CM alone, and all of the meta-analyses found a higher total effective rate in the combined treatment group than in the CM group, with all demonstrating low heterogeneity (*I*^2^ = 0%). Regarding the quality of evidence, two meta-analyses (23, 30) showed a “moderate” quality of evidence for the results, and six (10, 24, 26, 31, 33, 34) were determined as “low” quality. The study with the largest sample size included 24 RCTs (31), with a total of 3,371 patients (RR = 1.20, 95% CI = 1.16 to 1.23, *p* < 0.00001, *I*^2^ = 0%, fixed effect model).

#### Left ventricular end-diastolic dimension

Seven systematic reviews (10, 12, 24, 25, 28, 30, 33) evaluated the potential benefits of QSYQ combined with CM on LVEDD, and all of the meta-analyses showed that QSYQ combined with CM could improve LVEDD better than CM alone. However, most results(6/7, 85.71%) (10, 12, 24, 28, 30, 33) showed high heterogeneity. All studies had a “low” quality of evidence for the results. The study with the largest sample size included 14 RCTs (12), with a total of 1,665 patients (SMD = –1.34, 95% CI = –1.87 to –0.80, *p* < 0.00001, *I*^2^ = 96%, random effect model).

#### Left ventricular end-systolic internal diameter

Six systematic reviews (10, 12, 24, 25, 28, 32) evaluated the potential benefits of QSYQ combined with CM on LVESD, and all of the meta-analyses showed that QSYQ combined with CM improved LVESD better than CM alone. The results of three studies (10, 12, 32) demonstrated high heterogeneity and the quality of evidence was rated as “moderate” for one outcome (28) and “low” for the rest. The study with the largest sample size included 13 RCTs (12), with a total of 1,592 patients (SMD = –0.60, 95%CI = –1.14 to –0.05, *p* < 0.00001, *I*^2^ = 96%, random effect model).

#### Six-minutes walking distance

Ten systematic reviews (10, 12, 23, 27, 29–34) showed that QSYQ combined with CM treatment was better than CM in improving the 6MWD of patients with CHF, but most of the results (9/10, 90%) (10, 12, 23, 27, 29–31, 33, 34) were significantly heterogeneous. The quality of evidence was “low” to “very low.” The study with the largest sample size included 14 RCTs (31), comprising a total of 2,197 patients (MD = 45.08, 95% CI = 43.09–47.08, *p* < 0.00001, *I*^2^ = 99%, random effect model).

#### Brain natriuretic peptide

Nine systematic reviews (10, 12, 23, 24, 27, 29–32) evaluated the potential benefits of QSYQ combined with CM on decreased BNP. Among the eight systematic reviews (10, 12, 24, 27, 29–32), QSYQ combined with CM decreased BNP better than CM alone. The study with the largest sample size included 17 RCTs (10), comprising a total of 1,762 patients (SMD = –2.26, 95% CI –2.89 to –1.63, *p* < 0.00001, *I*^2^ = 96%, random effect model). However, there was no significant difference was observed in one study (23) (5 RCTs, SMD = 194.85, 95% CI = –52.91 to 442.61, *P* < 0.001, *I*^2^ = 99%, random effect model, “low” quality of evidence).

#### N-terminal pro-brain natriuretic peptide

Two systematic reviews (12, 34) evaluated the potential benefits of QSYQ combined with CM on decreasing NT-proBNP, and all meta-analyses showed that QSYQ combined with CM decreased NT-proBNP better than CM alone. However, there was significant heterogeneity in all of these results, with one study (34) having “low” quality of evidence and another (12) with “very low” quality of evidence. The study with the largest sample size included 11 RCTs (34), comprising a total of 1,328 patients (SMD = –1.45, 95% CI = –2.00 to –1.14, *p* < 0.00001, *I*^2^ = 95%, random effect model).

### Safety of Qishen Yiqi for chronic heart failure

Of the 14 included systematic reviews, 11 (11/14, 78.57%) (10, 23–27, 29, 31–34) reported adverse effects associated with QSYQ, which mainly included hypotension, dry cough, dizziness and headache. Overall, the results suggested that the combination of QSYQ adjuvant therapy with CM treatment was safe and did not significantly increase the risk of adverse events compared with CM only. However, it should be noted that most of the original RCTs included in the 10 systematic reviews (10, 23–25, 27, 29, 31–34) did not report adverse reactions.

## Discussion

In China, many patients with CHF undergo adjunctive treatment with Chinese proprietary medicines, such as QSYQ, due to unsatisfactory treatment of symptoms, reduced quality of life, or side effects of conventional treatment (35). This has aroused the interest of investigators, and many related RCTs were conducted. Previous works demonstrated that the combination of QSYQ appeared to be more effective and had a good safety profile in the treatment of CHF in addition to conventional treatment (36). Related meta-analyses (10, 12) have also been published more frequently, but the results remained controversial regarding the clinical effectiveness and safety of QSYQ for CHF. Therefore, we conducted this review, retrieved relevant systematic reviews of all corresponding RCTs of QSYQ for CHF, evaluated the methodological quality using the AMSTAR-2 tool, and GRADE evaluated the level of evidence.

### Summary of findings

In this present review, the evidence on the efficacy and safety of QSYQ for the treatment of CHF was derived from 14 systematic reviews. Overall, the available evidence strongly suggested that QSYQ was effective as an adjunctive treatment for CHF, as evidenced by greater benefits in improving cardiac function (e.g., increasing LVEF and reducing LVEDD and LVESD), increasing the total effective rate and 6MWD, and decreasing NT-proBNP. In terms of decreasing BNP, while eight meta-analyses showed QSYQ to be effective, one meta-analysis (23) reported no advantage compared with controls. No serious adverse events were associated with QSYQ. However, the overall methodological quality and data reporting quality of the original RCTs included in these systematic reviews were generally poor, and the lack of large-sample, multicenter, placebo studies contributed to the inability of almost all included systematic reviews to draw firm and reliable conclusions about the efficacy and safety of QSYQ in CHF. In addition, the methodological and evidentiary quality of most systematic reviews was unsatisfactory, as shown by the results of the AMSTAR-2, PRISMA 2020, ROBIS and GRADE assessments. Therefore, there is an urgent need for future RCTs and systematic reviews to further improve the methodological design to accurately determine the true effectiveness and safety of QSYQ for the treatment of CHF.

High-quality systematic reviews can provide clinicians, patients, and other decision-makers with a reliable scientific basis (37). The methodological quality of the systematic review was assessed using the AMSTAR-2 tool, and our results indicated that the methodological quality of these systematic reviews was “critical low.” The following are the potential considerations for improving the quality of future studies: (1) none of the systematic reviews included in the QSYQ for CHF were preregistered prior to the study initiation, which was also reflected in the original RCT. Systematic reviews or RCTs are often performed with substantial financial support; thus, duplicate or similar studies might waste resources, and registration would allow researchers to check if similar topics already exist or are “in progress” at the planning stage of the study to determine if it is necessary to proceed with a similar project (38). In addition, the transparency of research, accuracy and completeness of test methods should be improved once the results are published, thereby reducing selective reporting of results and publication bias and improving the authenticity of the research (39). It is worth noting that clinical trial registration is an ethical imperative for medical research, as well as a responsibility and obligation of trial investigators (40). Therefore, we call for future systematic reviews or RCTs on the effectiveness of QSYQ in CHF to be preregistered on relevant websites. (2) All of the included reviews selected only RCTs but did not explain the specific reasons for the choice of study type. Although RCTs are the gold standard for assessing new drugs, systematic reviews of non-randomized intervention studies can also complement their role when there are few RCTs, missing outcome indicators and insufficient statistical effects (41). In addition to justifying the choice of study type, it is equally important to justify the reasons for the choice. (3) A high-quality systematic review requires a thorough, objective and reproducible search and screening of relevant studies after developing a research strategy and exhaustive inclusion criteria (42). However, none of the meta-analyses included in this overview searched gray literature. In addition, most of the systematic reviews did not present the full search strategy for all databases and websites in detail, including the filters and qualifiers used, and some studies did not specify a screening process in which at least two researchers independently screened the literature and extracted the data, which reduced the rigor and reproducibility of the corresponding studies. (4) Most of the reviews did not investigate the potential impact of the risk of bias of the inclusion of original RCTs on the meta-analysis results by subgroup analysis or sensitivity analysis. In the case of including only high-quality RCTs, there may be little discussion of the potential impact of bias on outcomes. However, none of the RCTs on QSYQ for CHF were of high quality, indicating the need to assess the impact of the risk of bias in RCTs on the review results. (5) Another important finding was that most of the authors of the systematic review (11/14, 78.57%) did not report all sources of potential conflicts of interest, which also contributed to the low quality of the methodology. Several lines of evidence (43) have demonstrated that pharmaceutical company-funded systematic reviews were more likely to yield effective interventions than unfunded studies and investigators should report the direct source of funding even if they do not receive funding but still have a relationship with the company whose product is involved in the systematic evaluation. Similar to the AMSTAR-2results, the PRISMA 2020 evaluation showed that the included systematic reviews also had the deficiencies mentioned above. The risk of bias assessment on the results ROBIS scale indicated that all systematic reviews were at high risk of bias. Further analysis showed that inadequate interpretation of risk of bias, risk of study identification and selection bias and inadequate assessment of publication bias were the main factors contributing to the high risk of bias.

The present overview assessed the quality of evidence of systematic reviews using the GRADE system. Our results showed that most outcome indicators (e.g., LVEF, total effective rate, LVEDD, LVESD, 6MWD, and NT-proBNP) indicated QSYQ was beneficial as an adjunctive treatment for patients with CHF. However, it is noteworthy that these evidence quality grades ranged from “very low” to “moderate.” Risk of bias, publication bias, and inconsistency were the primary reasons for the low quality of the evidence. The original RCTs included in the systematic reviews all had significant bias in their trial design, such as unclear study randomization schemes, lack of allocation concealment information, and failure to implement blinding. Therefore, future RCTs should strictly follow the “CONSORT Extension for Chinese Herbal Medicine Formulas 2017” statement (44) to further standardize their design in terms of random allocation, allocation concealment, and blinding. In addition, another factor leading to lower quality of evidence was inconsistency, with large heterogeneity of outcome indicators in most studies. Future meta-analyses should conduct subgroup analyses and sensitivity analyses of the more heterogeneous outcome indicators to identify sources of heterogeneity, and if the heterogeneity still cannot be reduced, descriptive analyses might be considered. Moreover, the lack of description of how sample sizes were determined in the original RCTs included in the systematic reviews and the small sample size reduced the precision, contributing to the reduced quality of evidence. Lastly, we also noted a lack of focus on the impact of QSYQ on reducing the morbidity, mortality and readmission rates of CHF patients in the RCTs. CHF is the end stage of various heart diseases with relatively high annual mortality rates; therefore, reducing mortality and readmission rates are the endpoint goals in the treatment of CHF (9), and future studies should focus on evaluating long-term efficacy indicators.

Over the past decades, several *in vivo* studies have attempted to elucidate the mechanism of action of QSYQ in the treatment of heart failure (45). In a mouse model of high-fat diet-induced heart failure with preserved ejection fraction (HFpEF), it was observed that QSYQ significantly improved cardiac function and myocardial remodeling in mice, possibly through the inhibition of microvascular endothelial inflammation and activation of the NO-cGMP-PKG pathway (46). In addition, it was also reported that, in coronary artery ligation-induced ischemic CHF rats, QSYQ intervention reduced myocardial infarct size and apoptosis and improved myocardial fibrosis (47). Interestingly, it was found that QSYQ also prevented doxorubicin-induced cardiotoxicity in mice, which may be closely related to enhanced cardiac angiogenesis (48). Altogether, the above preclinical studies suggest that the mechanism of action of QSYQ in the treatment of heart failure might be mediated through multiple targets and pathways.

The safety of Chinese medicines has been a widespread concern, and safety is an important outcome indicator in clinical studies of interventions (49). However, three systematic reviews (12, 28, 30) did not report adverse reactions associated with QSYQ. Although the conclusions of the original RCTs included in the meta-analysis indicated that QSYQ was safe with no major adverse events, it is important to note that these RCTs were vague in their descriptions of adverse events that occurred during the study, focused only on the discomforts exhibited, and lacked biochemical testing indicators. For instance, chronic liver and kidney damage was a frequently reported adverse effect of Chinese medicines (49), but patients with mild symptoms might not have reported these manifestations or felt the need to inform the investigators, thus unless proper serum biomarkers and abdominal ultrasounds are performed, these conditions might have been under-reported. Therefore, we suggest that future studies should adequately describe the specific methods and potential basis for QSYQ safety assessment. Details of all adverse events (e.g., time of occurrence, number or frequency, severity, the number of cases withdrawn, and/or dose reduction) should be reported. Lastly, the underlying cause or potential trigger should be discussed for any adverse events.

### Limitations

This is the first overview to examine the quality of evidence for the safety and efficacy of QSYQ in CHF patients using the AMSTAR-2, PRISMA 2020, ROBIS, and GRADE approaches. However, there were some limitations to our study. First, only studies published in English and Chinese were included in this study; considering that Chinese proprietary medicine is also popular in Asian countries, such as Korea and Japan, the inclusion of relevant systematic reviews might have provided new insights into the study findings. Second, this review did not conduct any quantitative analysis, which might have led to biased conclusions. Third, most of the systematic reviews included in this study were of poor quality, and all RCTs were conducted in China, which reduced the credibility of the evidence reported.

## Conclusion

Despite the reported efficacy and safety of QSYQ, current evidence limits our ability to confirm the benefits of QSYQ in CHF, given the poor methodological quality and low quality of evidence in most of the investigated systematic reviews. Thus, better-designed and high-quality clinical studies and systematic reviews are still needed to provide clear indications about the clinical significance of QSYQ in CHF.

## Data availability statement

The original contributions presented in this study are included in the article/Supplementary material, further inquiries can be directed to the corresponding authors.

## Author contributions

YL, DW, and WC designed this study. DW and JY performed the search. XY and YW collected the data. JC, XY, and YW rechecked the data. JC, YL, and WC performed the analysis. WC and YW drafted the manuscript. DW and YL revised the manuscript. All authors read and approved the final manuscript.

## Footnotes

1
https://osf.io/

## Abbreviations

6MWD: 6-min walking distance
RCTs: randomized controlled trials
AMSTAR 2: A Measurement Tool to Assess Systematic Reviews 2
ACE-Is: angiotensin-converting enzyme inhibitors
BNP: B-type brain natriuretic peptide
CHF: chronic heart failure
CM: conventional medicine
GRADE: the Grades of Recommendation, Assessment, Development, and Evaluation
HFpEF: heart failure with preserved ejection fraction
LVEF: left ventricular ejection fraction
LVEDD: left ventricular end-diastolic dimension
LVESD: left ventricular end-systolic internal diameter
NT-proBNP: N-terminal pro-brain natriuretic peptide
QSYQ: Qishen Yiqi.
