# Supplementary material for: The role of Qishen Yiqi dripping pills in treating chronic heart failure: An overview of systematic reviews and meta-analyses
Source: Front Cardiovasc Med. 2022 Oct 24;9:1001072. doi: 10.3389/fcvm.2022.1001072 (PMC9637556; doi:10.3389/fcvm.2022.1001072)
Supplement: Supplementary file 1 [file Data_Sheet_1.docx]

## Supplementary Material S1. Search strategies in databases

## **1.1 China National Knowledge Infrastructure**

The database search in CNKI was carried out on May 03, 2022, and a total of 20 studies were found.

**search strategy**

SU=('心力衰竭'+'心衰'+'心功能不全')*'芪参益气滴丸' *('系统评价'+'Meta'+'荟萃分析')


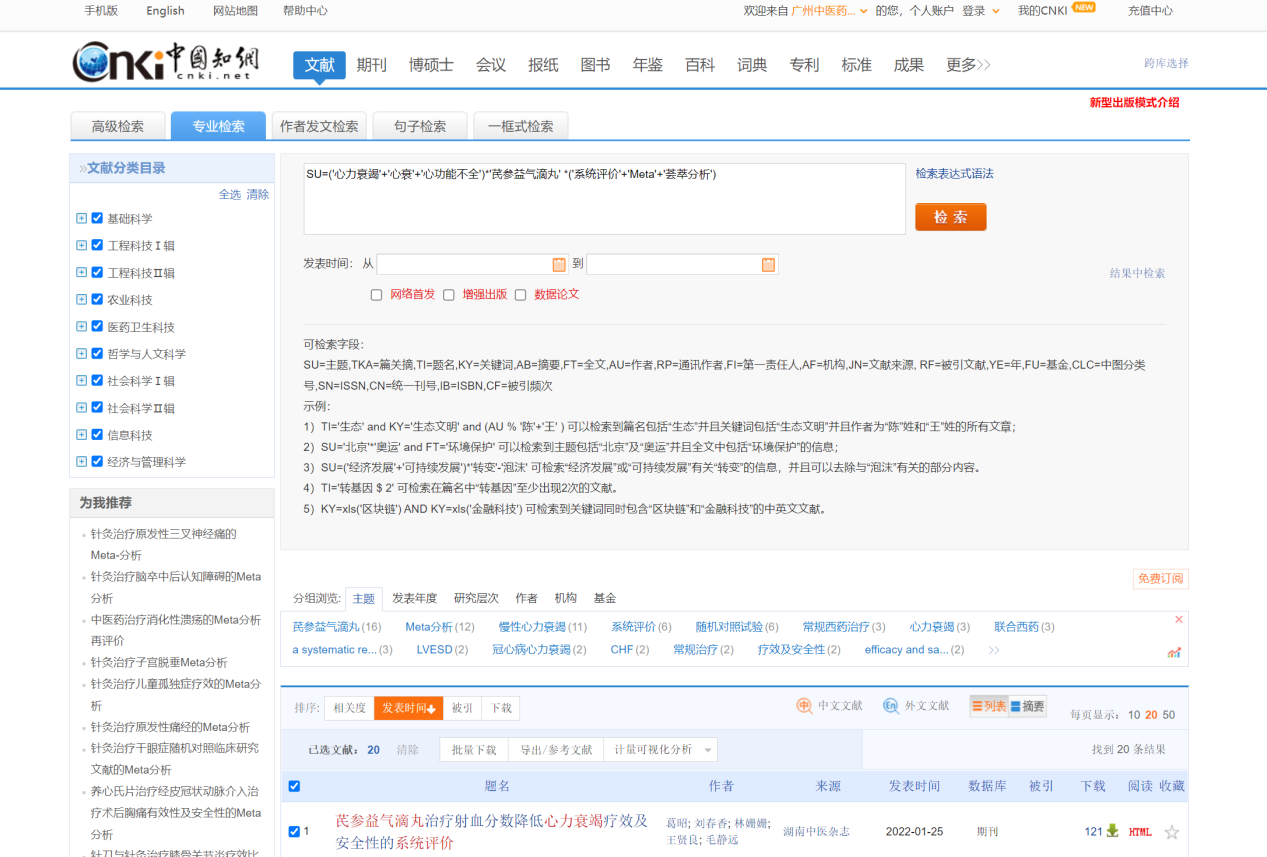


[1]葛昭,刘春香,林姗姗,王贤良,毛静远.芪参益气滴丸治疗射血分数降低心力衰竭疗效及安全性的系统评价[J].湖南中医杂志,2022,38(01):115-123.DOI:10.16808/j.cnki.issn1003-7705.2022.01.034.

[2]牛振超,林文勇,李益萍,强婷婷,王肖龙.芪参益气滴丸辨证施治对慢性心力衰竭疗效影响的Meta分析[J].药物评价研究,2021,44(05):1076-1087.

[3]林姗姗. 慢性心力衰竭中医诊疗指南的制定研究[D].天津中医药大学,2021.DOI:10.27368/d.cnki.gtzyy.2021.000338.

[4]Wang Mengxi,Shan Yiwen,Wu Chenjie,Cao Peihua,Sun Weixin,Han Jie,Shen Le,Chen Jiandong,Yu Peng,Chen Xiaohu. Efficacy and Safety of Qishen Yiqi Dripping Pill for Heart Failure With Preserved Ejection Fraction: A Systematic Review and Meta-Analysis

[J]. Frontiers in Pharmacology,2021,11.

[5]Chen Lisheng,Wang Ruilin,Liu Honghong,Wei Shizhang,Jing Manyi,Wang Min,Zhao Yanling,Uddin Md. Sahab. Clinical Efficacy and Safety of Qishen Yiqi Dropping Pill Combined with Conventional Western Medicine in the Treatment of Chronic Heart Failure: A Systematic Review and Meta-Analysis[J]. Evidence-Based Complementary and Alternative Medicine,2021,2021.

[6]张秀文,戴雁彦,王宗亮,杨昊昕,辛玉.芪参益气滴丸联合曲美他嗪治疗慢性心力衰竭的Meta分析[J].世界中西医结合杂志,2021,16(01):23-28+33.DOI:10.13935/j.cnki.sjzx.210105.

[7]Guan Hui,Dai Guohua,Ren Lili,Gao Wulin,Fu Haoran,Zhao Zepeng,Liu Xin,Li Jue. Efficacy and safety of Qishen Yiqi dripping pills as a complementary treatment for Heart Failure: A protocol of updated systematic review and meta-analysis[J]. Medicine,2021,100(2).

[8]李雪靖,张慧玲,王晓丽,高胜男,刘洋,刘国强.芪参益气滴丸治疗慢性心力衰竭的循证药物经济学评价[J].中国医院用药评价与分析,2020,20(12):1472-1477+1482.DOI:10.14009/j.issn.1672-2124.2020.12.017.

[9]Wang H, Li L, Qing X, Zhang S, Li S. Efficacy of Qishen Yiqi Drop Pill for Chronic Heart Failure: An Updated Meta-Analysis of 85 Studies. Cardiovasc Ther. 2020 Sep 22;2020:8138764. doi: 10.1155/2020/8138764. PMID: 33042225; PMCID: PMC7530480.

[10]谢锋,段广靖,王斌,卫培峰,陈琳,李敏.芪参益气滴丸联合曲美他嗪治疗慢性心力衰竭的meta分析[J].海南医学院学报,2021,27(09):689-694+700.DOI:10.13210/j.cnki.jhmu.20200814.004.

[11]樊根豪,邢作英,陈召起,张孟孟,胡宇才,赵安社,刘梦琳,王永霞.芪参益气滴丸治疗冠心病心力衰竭的系统评价[J].中国中医基础医学杂志,2020,26(07):932-935+997.

[12]郭娇,刘新灿,孙天福.芪参益气滴丸联合西药治疗慢性心力衰竭的临床疗效和安全性分析[J].中国循证心血管医学杂志,2020,12(05):519-524.

[13]章轶立,王娟,李园,赵慧辉,刘俊杰,王伟.芪参益气滴丸治疗慢性心力衰竭(气虚血瘀证)的Meta分析[J].中国实验方剂学杂志,2019,25(21):162-169.DOI:10.13422/j.cnki.syfjx.20191447.

[14]单秋月,张闻,吕露,李凌艳,孙鹤,郭治昕.用芪参益气滴丸抑制慢性心力衰竭患者心室重构效果的Meta分析[J].当代医药论丛,2017,15(11):71-73.

[15]田野,顾健霞.芪参益气滴丸治疗冠心病心力衰竭的Meta分析[J].中国中医急症,2016,25(09):1725-1727+1742.

[16]刘军刚,顾万红,刘效栓,李喜香,黄清杰.芪参益气滴丸治疗慢性心力衰竭的Meta分析[J].中国新药与临床杂志,2014,33(03):189-195.

[17]高长春,徐国良,秦玲.芪参益气滴丸治疗慢性充血性心力衰竭有效性及安全性的Meta分析[J].中国中医急症,2014,23(02):232-234.

[18]曲凤,邢冬梅,郑文科,田盈,李焱,康立源.芪参益气滴丸治疗缺血性心力衰竭的系统评价[J].中国实验方剂学杂志,2014,20(03):213-218.

[19]王拴虎,毛静远,侯雅竹,王家莹,王贤良,李志君.西药常规加用芪参益气滴丸治疗慢性心力衰竭随机对照试验的系统评价[J].中国中西医结合杂志,2013,33(11):1468-1475.

[20]裴英豪,朱翠玲,朱明军,闫奎坡,陈晓旭.芪参益气滴丸治疗慢性心力衰竭的疗效及安全性系统评价[J].中国中医急症,2013,22(09):1472-1475.

## **1.2 China Science and Technology Journal Database**

The database search in VIP was carried out on May 03, 2022, and a total of 15 studies were found.

**search strategy**

M=(心力衰竭 OR 心衰 OR 心功能不全) AND M=芪参益气滴丸 AND M=(Meta OR 系统评价 OR 荟萃分析)


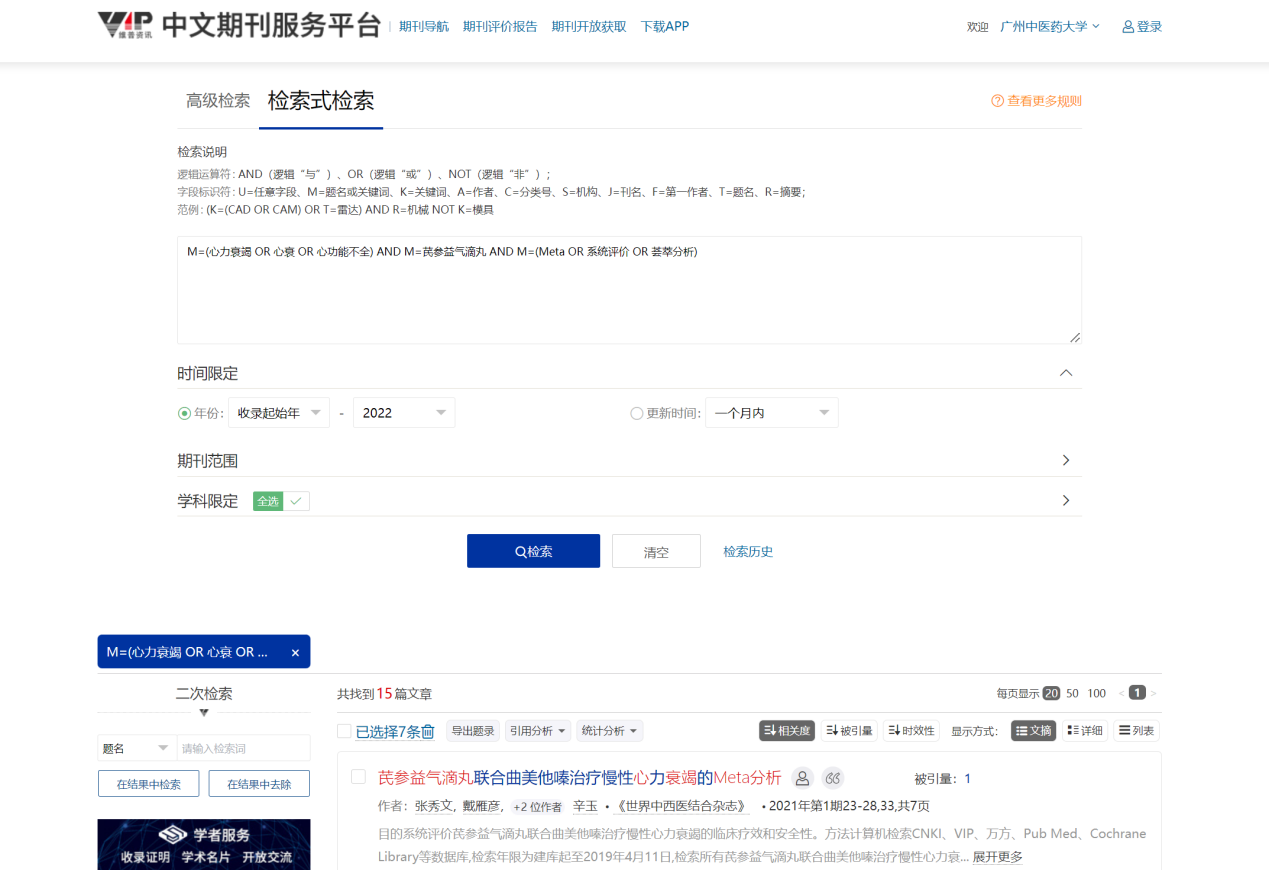


[1]裴英豪,朱翠玲,朱明军,闫奎坡,陈晓旭.芪参益气滴丸治疗慢性心力衰竭的疗效及安全性系统评价[J].中国中医急症,2013,22(9):1472-1475.

[2]王拴虎,毛静远,侯雅竹,王家莹,王贤良,李志君.西药常规加用芪参益气滴丸治疗慢性心力衰竭随机对照试验的系统评价[J].中国中西医结合杂志,2013,33(11):1468-1475.

[3]曲凤,邢冬梅,郑文科,田盈,李焱,康立源.芪参益气滴丸治疗缺血性心力衰竭的系统评价[J].中国实验方剂学杂志,2014,20(3):213-218.

[4]高长春,徐国良,秦玲.芪参益气滴丸治疗慢性充血性心力衰竭有效性及安全性的Meta分析[J].中国中医急症,2014,23(2):232-234.

[5]刘军刚,顾万红,刘效栓,李喜香,黄清杰.芪参益气滴丸治疗慢性心力衰竭的Meta分析[J].中国新药与临床杂志,2014,33(3):189-195.

[6]田野,顾健霞.芪参益气滴丸治疗冠心病心力衰竭的Meta分析[J].中国中医急症,2016,25(9):1725-1727+1742.

[7]单秋月,张闻,吕露,李凌艳,孙鹤,郭治昕.用芪参益气滴丸抑制慢性心力衰竭患者心室重构效果的Meta分析[J].当代医药论丛,2017,15(11):71-73.

[8]郭娇,刘新灿,孙天福.芪参益气滴丸联合西药治疗慢性心力衰竭的临床疗效和安全性分析[J].中国循证心血管医学杂志,2020,12(5):519-524.

[9]樊根豪,邢作英,陈召起,张孟孟,胡宇才,赵安社,刘梦琳,王永霞.芪参益气滴丸治疗冠心病心力衰竭的系统评价[J].中国中医基础医学杂志,2020,26(7):932-935+997.

[10]李雪靖,张慧玲,王晓丽,高胜男,刘洋,刘国强.芪参益气滴丸治疗慢性心力衰竭的循证药物经济学评价[J].中国医院用药评价与分析,2020,20(12):1472-1477+1482.

[11]张秀文,戴雁彦,王宗亮,杨昊昕,辛玉.芪参益气滴丸联合曲美他嗪治疗慢性心力衰竭的Meta分析[J].世界中西医结合杂志,2021,16(1):23-28+33.

[12]谢锋,段广靖,王斌,卫培峰,陈琳,李敏.芪参益气滴丸联合曲美他嗪治疗慢性心力衰竭的meta分析[J].海南医学院学报,2021,27(9):689-694+700.

[13]牛振超,林文勇,李益萍,强婷婷,王肖龙.芪参益气滴丸辨证施治对慢性心力衰竭疗效影响的Meta分析[J].药物评价研究,2021,44(5):1076-1087.

[14]葛昭,刘春香,林姗姗,王贤良,毛静远.芪参益气滴丸治疗射血分数降低心力衰竭疗效及安全性的系统评价[J].湖南中医杂志,2022,38(1):115-123.

[15]章轶立,王娟,李园,赵慧辉,刘俊杰,王伟.芪参益气滴丸治疗慢性心力衰竭(气虚血瘀证)的Meta分析[J].中国实验方剂学杂志,2019(21):162-169.

## **1.3 Wan Fang**

The database search in Wan Fang was carried out on May 03, 2022, and a total of 20 studies were found.

**search strategy**

主题:("心力衰竭" OR "心衰" OR "心功能不全") and 主题:("芪参益气滴丸")and 主题:("Meta" OR "系统评价" OR "荟萃分析")


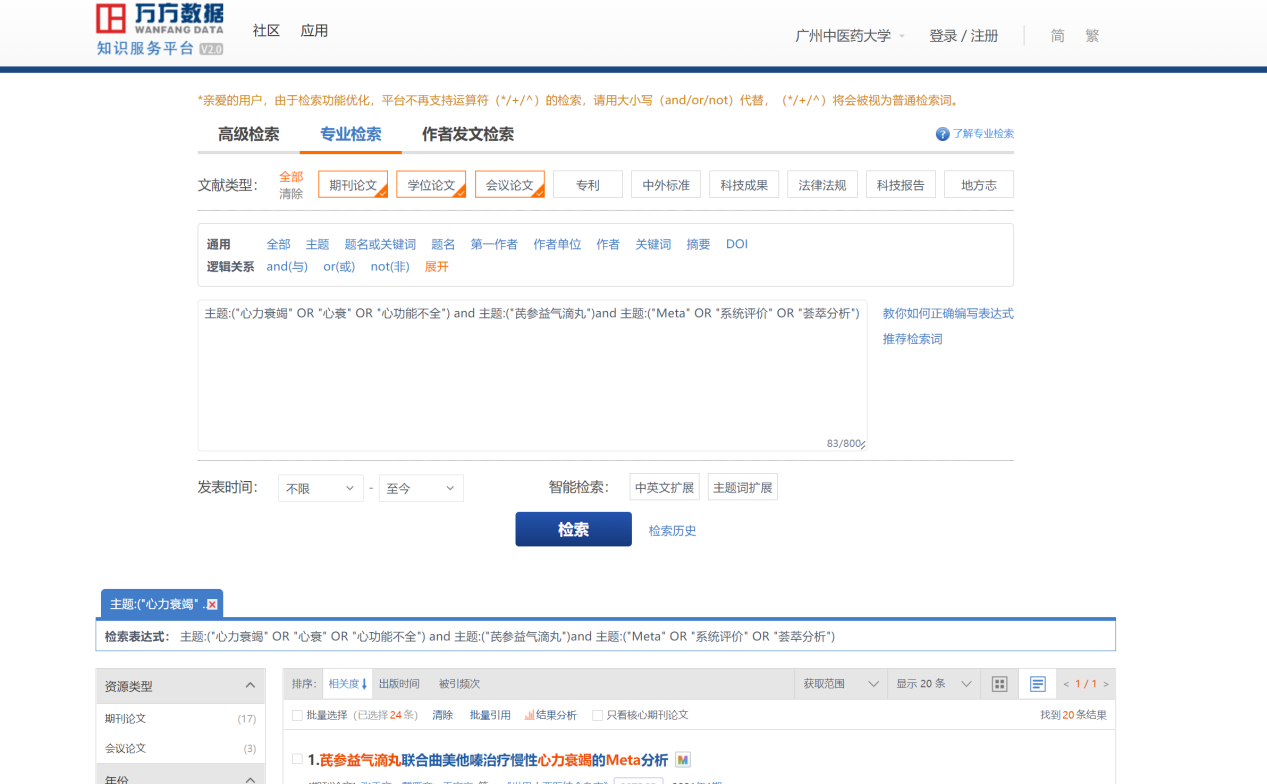


[1] 张秀文,戴雁彦,王宗亮,等. 芪参益气滴丸联合曲美他嗪治疗慢性心力衰竭的Meta分析[J]. 世界中西医结合杂志,2021,16(1):23-28,33. DOI:10.13935/j.cnki.sjzx.210105.

[2] 谢锋,段广靖,王斌,等. 芪参益气滴丸联合曲美他嗪治疗慢性心力衰竭的meta分析[J]. 海南医学院学报,2021,27(9):689-694,700. DOI:10.13210/j.cnki.jhmu.20200814.004.

[3] 田野,顾健霞. 芪参益气滴丸治疗冠心病心力衰竭的Meta分析[J]. 中国中医急症,2016,25(9):1725-1727,1742. DOI:10.3969/j.issn.1004-745X.2016.09.025.

[4] 牛振超,林文勇,李益萍,等. 芪参益气滴丸辨证施治对慢性心力衰竭疗效影响的Meta分析[J]. 药物评价研究,2021,44(5):1076-1087. DOI:10.7501/j.issn.1674-6376.2021.05.027.

[5] 单秋月,张闻,吕露,等. 用芪参益气滴丸抑制慢性心力衰竭患者心室重构效果的Meta分析[J]. 当代医药论丛,2017,15(11):71-73.

[6] 高长春,徐国良,秦玲. 芪参益气滴丸治疗慢性充血性心力衰竭有效性及安全性的Meta分析[J]. 中国中医急症,2014,23(2):232-234. DOI:10.3969/j.issn.1004-745X.2014.02.018.

[7] 樊根豪,邢作英,陈召起,等. 芪参益气滴丸治疗冠心病心力衰竭的系统评价[J]. 中国中医基础医学杂志,2020,26(7):932-935,997. DOI:10.3969/j.issn.1006-3250.2020.07.022.

[8] 王拴虎,毛静远,侯雅竹,等. 西药常规加用芪参益气滴丸治疗慢性心力衰竭随机对照试验的系统评价[J]. 中国中西医结合杂志,2013,33(11):1468-1475. DOI:10.7661/CJIM.2013.11.1468.

[9] 葛昭,刘春香,林姗姗,等. 芪参益气滴丸治疗射血分数降低心力衰竭疗效及安全性的系统评价[J]. 湖南中医杂志,2022,38(1):115-123. DOI:10.16808/j.cnki.issn1003-7705.2022.01.034.

[10] 郭娇,刘新灿,孙天福. 芪参益气滴丸联合西药治疗慢性心力衰竭的临床疗效和安全性分析[J]. 中国循证心血管医学杂志,2020,12(5):519-524. DOI:10.3969/j.issn.1674-4055.2020.05.03.

[11] 李雪靖,张慧玲,王晓丽,等. 芪参益气滴丸治疗慢性心力衰竭的循证药物经济学评价[J]. 中国医院用药评价与分析,2020,20(12):1472-1477,1482. DOI:10.14009/j.issn.1672-2124.2020.12.017.

[12] 裴英豪,朱翠玲,朱明军,等. 芪参益气滴丸治疗慢性心力衰竭的疗效及安全性系统评价[J]. 中国中医急症,2013,22(9):1472-1475. DOI:10.3969/j.issn.1004-745X.2013.09.007.

[13] 章轶立,王娟,李园,等. 芪参益气滴丸治疗慢性心力衰竭(气虚血瘀证)的Meta分析[J]. 中国实验方剂学杂志,2019,25(21):162-169. DOI:10.13422/j.cnki.syfjx.20191447.

[14] 代倩倩,石兆峰,胡嘉元,等. 芪参益气滴丸联合西药治疗对经皮冠状动脉介入术后不良心血管事件及生活质量影响的Meta分析[J]. 中国中药杂志,2021,46(6):1498-1510. DOI:10.19540/j.cnki.cjcmm.20200618.501.

[15] 王越,戴小华. 芪参益气滴丸治疗慢性心力衰竭疗效的Meta分析[C]. //中华中医药学会心病分会2015年学术会议论文集. 2015:216-223.

[16] 陈慧,李春,石天娇,等. 芪参益气滴丸对心衰大鼠RAAS系统的实验研究[J]. 北京中医药大学学报,2014,37(8):538-542. DOI:10.3969/j.issn.1006-2157.2014.08.009.

[17] 曲凤,邢冬梅,郑文科,等. 芪参益气滴丸治疗缺血性心力衰竭的系统评价[J]. 中国实验方剂学杂志,2014,20(3):213-218. DOI:10.11653/syfj2014030213.

[18] 戴小华,王越. 芪参益气滴丸治疗慢性心力衰竭疗效的Meta分析[C]. //第17届中国南方国际心血管病学术会议论文集. 2015:182-183.

[19] 樊根豪,王永霞,朱明军,等. 芪参益气滴丸治疗冠心病心力衰竭的系统评价[C]. //第十四届南方中医心血管病研讨会论文集. 2018:193-193.

[20] 朱方敏. 西药常规加用芪参益气滴丸治疗慢性心力衰竭随机对照试验的系统评价分析[J]. 健康之路,2014(8):324-325. DOI:10.3969/j.issn.1671-8801.2014.08.537.

## **1.4 PubMed**

The database search in PubMed was carried out on May 03, 2022, and a total of 7 studies were found.

**search strategy**

| Search number | Query | Search Details | Results | Time |
| --- | --- | --- | --- | --- |
| 4 | (((((((((((((((("Heart Failure"[Mesh]) OR (Cardiac Failure)) OR (Heart Decompensation)) OR (Decompensation, Heart)) OR (Heart Failure, Right-Sided)) OR (Heart Failure, Right Sided)) OR (Right-Sided Heart Failure)) OR (Right Sided Heart Failure)) OR (Myocardial Failure)) OR (Congestive Heart Failure)) OR (Heart Failure, Congestive)) OR (Heart Failure, Left-Sided)) OR (Heart Failure, Left Sided)) OR (Left-Sided Heart Failure)) OR (Left Sided Heart Failure)) AND ((Qishen Yiqi Dripping Pill) OR (Qishen Yiqi))) AND ((Meta-Analysis) OR (system assesment)) | ("Heart Failure"[MeSH Terms] OR ("Heart Failure"[MeSH Terms] OR ("heart"[All Fields] AND "failure"[All Fields]) OR "Heart Failure"[All Fields] OR ("cardiac"[All Fields] AND "failure"[All Fields]) OR "cardiac failure"[All Fields]) OR ("Heart Failure"[MeSH Terms] OR ("heart"[All Fields] AND "failure"[All Fields]) OR "Heart Failure"[All Fields] OR ("heart"[All Fields] AND "decompensation"[All Fields]) OR "heart decompensation"[All Fields]) OR ("Heart Failure"[MeSH Terms] OR ("heart"[All Fields] AND "failure"[All Fields]) OR "Heart Failure"[All Fields] OR ("decompensation"[All Fields] AND "heart"[All Fields]) OR "decompensation heart"[All Fields]) OR ("Heart Failure"[MeSH Terms] OR ("heart"[All Fields] AND "failure"[All Fields]) OR "Heart Failure"[All Fields] OR ("heart"[All Fields] AND "failure"[All Fields] AND "right"[All Fields] AND "sided"[All Fields]) OR "heart failure right sided"[All Fields]) OR ("Heart Failure"[MeSH Terms] OR ("heart"[All Fields] AND "failure"[All Fields]) OR "Heart Failure"[All Fields] OR ("heart"[All Fields] AND "failure"[All Fields] AND "right"[All Fields] AND "sided"[All Fields]) OR "heart failure right sided"[All Fields]) OR ("Heart Failure"[MeSH Terms] OR ("heart"[All Fields] AND "failure"[All Fields]) OR "Heart Failure"[All Fields] OR ("right"[All Fields] AND "sided"[All Fields] AND "heart"[All Fields] AND "failure"[All Fields]) OR "right sided heart failure"[All Fields]) OR ("Heart Failure"[MeSH Terms] OR ("heart"[All Fields] AND "failure"[All Fields]) OR "Heart Failure"[All Fields] OR ("right"[All Fields] AND "sided"[All Fields] AND "heart"[All Fields] AND "failure"[All Fields]) OR "right sided heart failure"[All Fields]) OR ("Heart Failure"[MeSH Terms] OR ("heart"[All Fields] AND "failure"[All Fields]) OR "Heart Failure"[All Fields] OR ("myocardial"[All Fields] AND "failure"[All Fields]) OR "myocardial failure"[All Fields]) OR ("Heart Failure"[MeSH Terms] OR ("heart"[All Fields] AND "failure"[All Fields]) OR "Heart Failure"[All Fields] OR ("congestive"[All Fields] AND "heart"[All Fields] AND "failure"[All Fields]) OR "congestive heart failure"[All Fields]) OR ("Heart Failure"[MeSH Terms] OR ("heart"[All Fields] AND "failure"[All Fields]) OR "Heart Failure"[All Fields] OR ("heart"[All Fields] AND "failure"[All Fields] AND "congestive"[All Fields]) OR "heart failure congestive"[All Fields]) OR ("Heart Failure"[MeSH Terms] OR ("heart"[All Fields] AND "failure"[All Fields]) OR "Heart Failure"[All Fields] OR ("heart"[All Fields] AND "failure"[All Fields] AND "left"[All Fields] AND "sided"[All Fields]) OR "heart failure left sided"[All Fields]) OR ("Heart Failure"[MeSH Terms] OR ("heart"[All Fields] AND "failure"[All Fields]) OR "Heart Failure"[All Fields] OR ("heart"[All Fields] AND "failure"[All Fields] AND "left"[All Fields] AND "sided"[All Fields]) OR "heart failure left sided"[All Fields]) OR ("Heart Failure"[MeSH Terms] OR ("heart"[All Fields] AND "failure"[All Fields]) OR "Heart Failure"[All Fields] OR ("left"[All Fields] AND "sided"[All Fields] AND "heart"[All Fields] AND "failure"[All Fields]) OR "left sided heart failure"[All Fields]) OR ("Heart Failure"[MeSH Terms] OR ("heart"[All Fields] AND "failure"[All Fields]) OR "Heart Failure"[All Fields] OR ("left"[All Fields] AND "sided"[All Fields] AND "heart"[All Fields] AND "failure"[All Fields]) OR "left sided heart failure"[All Fields])) AND ((("qishen yiqi"[Supplementary Concept] OR "qishen yiqi"[All Fields]) AND ("dripped"[All Fields] OR "dripping"[All Fields] OR "drippings"[All Fields]) AND ("contraceptives, oral"[MeSH Terms] OR ("contraceptives"[All Fields] AND "oral"[All Fields]) OR "oral contraceptives"[All Fields] OR "pill"[All Fields])) OR ("qishen yiqi"[Supplementary Concept] OR "qishen yiqi"[All Fields])) AND ("meta analysis"[Publication Type] OR "meta analysis as topic"[MeSH Terms] OR "meta analysis"[All Fields] OR (("drug delivery systems"[MeSH Terms] OR ("drug"[All Fields] AND "delivery"[All Fields] AND "systems"[All Fields]) OR "drug delivery systems"[All Fields] OR "system"[All Fields] OR "system s"[All Fields] OR "systems"[All Fields]) AND ("assesed"[All Fields] OR "assesment"[All Fields] OR "assesments"[All Fields]))) |  | 23:04:27 |
| 3 | (Meta-Analysis) OR (system assesment) | "meta analysis"[Publication Type] OR "meta analysis as topic"[MeSH Terms] OR "meta analysis"[All Fields] OR (("drug delivery systems"[MeSH Terms] OR ("drug"[All Fields] AND "delivery"[All Fields] AND "systems"[All Fields]) OR "drug delivery systems"[All Fields] OR "system"[All Fields] OR "system s"[All Fields] OR "systems"[All Fields]) AND ("assesed"[All Fields] OR "assesment"[All Fields] OR "assesments"[All Fields])) | 243,000 | 23:04:15 |
| 2 | (Qishen Yiqi Dripping Pill) OR (Qishen Yiqi) | (("qishen yiqi"[Supplementary Concept] OR "qishen yiqi"[All Fields]) AND ("dripped"[All Fields] OR "dripping"[All Fields] OR "drippings"[All Fields]) AND ("contraceptives, oral"[MeSH Terms] OR ("contraceptives"[All Fields] AND "oral"[All Fields]) OR "oral contraceptives"[All Fields] OR "pill"[All Fields])) OR ("qishen yiqi"[Supplementary Concept] OR "qishen yiqi"[All Fields]) | 68 | 23:04:05 |
| 1 | (((((((((((((("Heart Failure"[Mesh]) OR (Cardiac Failure)) OR (Heart Decompensation)) OR (Decompensation, Heart)) OR (Heart Failure, Right-Sided)) OR (Heart Failure, Right Sided)) OR (Right-Sided Heart Failure)) OR (Right Sided Heart Failure)) OR (Myocardial Failure)) OR (Congestive Heart Failure)) OR (Heart Failure, Congestive)) OR (Heart Failure, Left-Sided)) OR (Heart Failure, Left Sided)) OR (Left-Sided Heart Failure)) OR (Left Sided Heart Failure) | "Heart Failure"[MeSH Terms] OR ("Heart Failure"[MeSH Terms] OR ("heart"[All Fields] AND "failure"[All Fields]) OR "Heart Failure"[All Fields] OR ("cardiac"[All Fields] AND "failure"[All Fields]) OR "cardiac failure"[All Fields]) OR ("Heart Failure"[MeSH Terms] OR ("heart"[All Fields] AND "failure"[All Fields]) OR "Heart Failure"[All Fields] OR ("heart"[All Fields] AND "decompensation"[All Fields]) OR "heart decompensation"[All Fields]) OR ("Heart Failure"[MeSH Terms] OR ("heart"[All Fields] AND "failure"[All Fields]) OR "Heart Failure"[All Fields] OR ("decompensation"[All Fields] AND "heart"[All Fields]) OR "decompensation heart"[All Fields]) OR ("Heart Failure"[MeSH Terms] OR ("heart"[All Fields] AND "failure"[All Fields]) OR "Heart Failure"[All Fields] OR ("heart"[All Fields] AND "failure"[All Fields] AND "right"[All Fields] AND "sided"[All Fields]) OR "heart failure right sided"[All Fields]) OR ("Heart Failure"[MeSH Terms] OR ("heart"[All Fields] AND "failure"[All Fields]) OR "Heart Failure"[All Fields] OR ("heart"[All Fields] AND "failure"[All Fields] AND "right"[All Fields] AND "sided"[All Fields]) OR "heart failure right sided"[All Fields]) OR ("Heart Failure"[MeSH Terms] OR ("heart"[All Fields] AND "failure"[All Fields]) OR "Heart Failure"[All Fields] OR ("right"[All Fields] AND "sided"[All Fields] AND "heart"[All Fields] AND "failure"[All Fields]) OR "right sided heart failure"[All Fields]) OR ("Heart Failure"[MeSH Terms] OR ("heart"[All Fields] AND "failure"[All Fields]) OR "Heart Failure"[All Fields] OR ("right"[All Fields] AND "sided"[All Fields] AND "heart"[All Fields] AND "failure"[All Fields]) OR "right sided heart failure"[All Fields]) OR ("Heart Failure"[MeSH Terms] OR ("heart"[All Fields] AND "failure"[All Fields]) OR "Heart Failure"[All Fields] OR ("myocardial"[All Fields] AND "failure"[All Fields]) OR "myocardial failure"[All Fields]) OR ("Heart Failure"[MeSH Terms] OR ("heart"[All Fields] AND "failure"[All Fields]) OR "Heart Failure"[All Fields] OR ("congestive"[All Fields] AND "heart"[All Fields] AND "failure"[All Fields]) OR "congestive heart failure"[All Fields]) OR ("Heart Failure"[MeSH Terms] OR ("heart"[All Fields] AND "failure"[All Fields]) OR "Heart Failure"[All Fields] OR ("heart"[All Fields] AND "failure"[All Fields] AND "congestive"[All Fields]) OR "heart failure congestive"[All Fields]) OR ("Heart Failure"[MeSH Terms] OR ("heart"[All Fields] AND "failure"[All Fields]) OR "Heart Failure"[All Fields] OR ("heart"[All Fields] AND "failure"[All Fields] AND "left"[All Fields] AND "sided"[All Fields]) OR "heart failure left sided"[All Fields]) OR ("Heart Failure"[MeSH Terms] OR ("heart"[All Fields] AND "failure"[All Fields]) OR "Heart Failure"[All Fields] OR ("heart"[All Fields] AND "failure"[All Fields] AND "left"[All Fields] AND "sided"[All Fields]) OR "heart failure left sided"[All Fields]) OR ("Heart Failure"[MeSH Terms] OR ("heart"[All Fields] AND "failure"[All Fields]) OR "Heart Failure"[All Fields] OR ("left"[All Fields] AND "sided"[All Fields] AND "heart"[All Fields] AND "failure"[All Fields]) OR "left sided heart failure"[All Fields]) OR ("Heart Failure"[MeSH Terms] OR ("heart"[All Fields] AND "failure"[All Fields]) OR "Heart Failure"[All Fields] OR ("left"[All Fields] AND "sided"[All Fields] AND "heart"[All Fields] AND "failure"[All Fields]) OR "left sided heart failure"[All Fields]) | 320,025 | 23:03:50 |


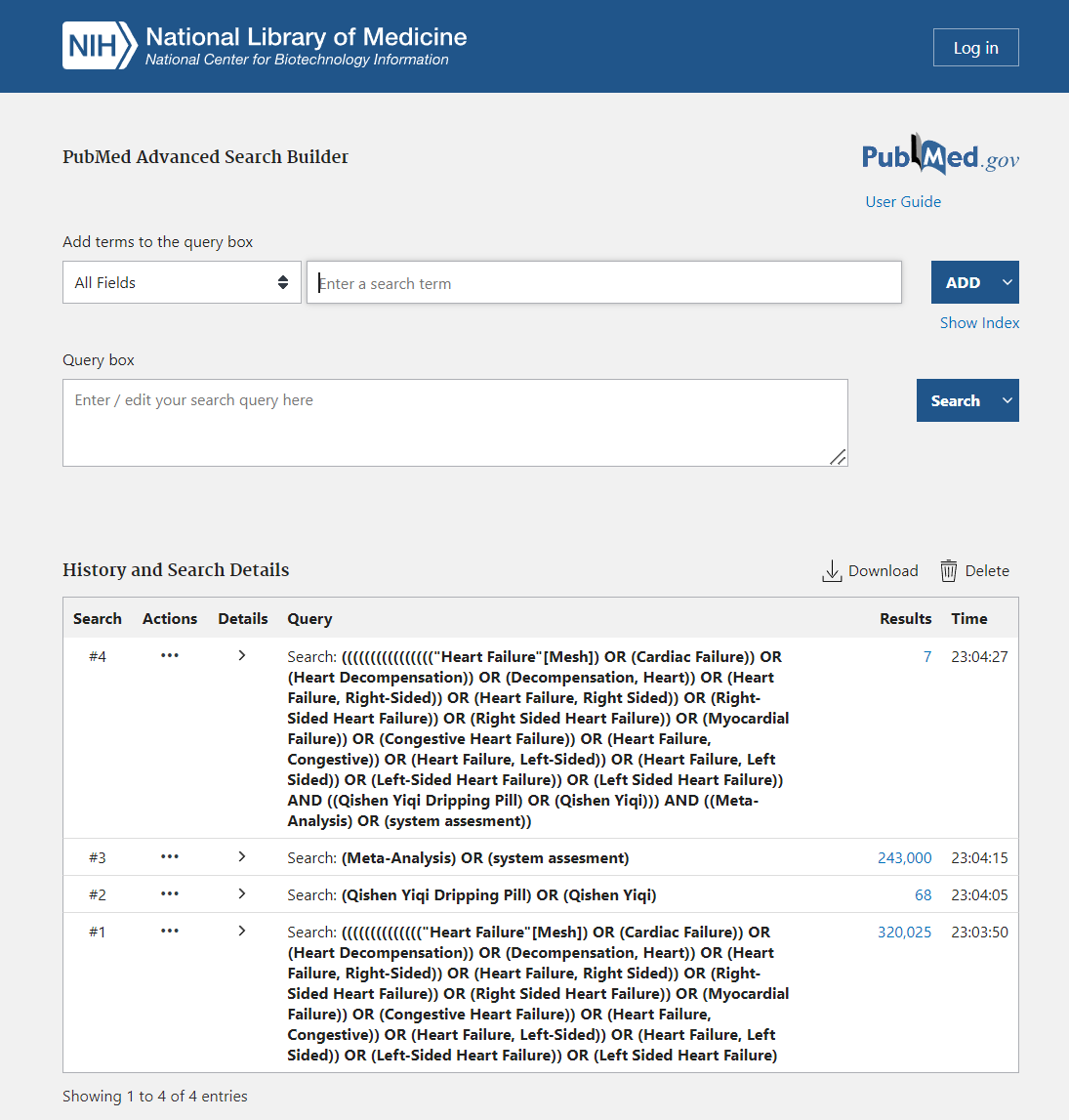


1. Wang H, Li L, Qing X, Zhang S, Li S. Efficacy of Qishen Yiqi Drop Pill for Chronic Heart Failure: An Updated Meta-Analysis of 85 Studies. Cardiovasc Ther. 2020 Sep 22;2020:8138764. doi: 10.1155/2020/8138764. PMID: 33042225; PMCID: PMC7530480.
2. Wang SH, Mao JY, Hou YZ, Wang JY, Wang XL, Li ZJ. [Routine western medicine treatment plus qishen yiqi dripping pill for treating patients with chronic heart failure: a systematic review of randomized control trials]. Zhongguo Zhong Xi Yi Jie He Za Zhi. 2013 Nov;33(11):1468-75. Chinese. PMID: 24483105.
3. Dai QQ, Shi ZF, Hu JY, Han SJ, Zhong CM, Guan MK, Tian GH, Shang HC. [Meta-analysis of effect of Qishen Yiqi Dripping Pills combined with Western medicine on adverse cardiovascular events and quality of life after percutaneous coronary intervention]. Zhongguo Zhong Yao Za Zhi. 2021 Mar;46(6):1498-1510. Chinese. doi: 10.19540/j.cnki.cjcmm.20200618.501. PMID: 33787149.
4. Guan H, Dai G, Ren L, Gao W, Fu H, Zhao Z, Liu X, Li J. Efficacy and safety of Qishen Yiqi dripping pills as a complementary treatment for Heart Failure: A protocol of updated systematic review and meta-analysis. Medicine (Baltimore). 2021 Jan 15;100(2):e24285. doi: 10.1097/MD.0000000000024285. PMID: 33466215; PMCID: PMC7808537.
5. Chen L, Wang R, Liu H, Wei S, Jing M, Wang M, Zhao Y. Clinical Efficacy and Safety of Qishen Yiqi Dropping Pill Combined with Conventional Western Medicine in the Treatment of Chronic Heart Failure: A Systematic Review and Meta-Analysis. Evid Based Complement Alternat Med. 2021 Feb 2;2021:6612653. doi: 10.1155/2021/6612653. PMID: 33603818; PMCID: PMC7872761.
6. Chang M, Cheng L, Shen Y, Zhang Y, Zhang Z, Hao P. Qishenyiqi dripping pill improves ventricular remodeling and function in patients with chronic heart failure: A pooled analysis. Medicine (Baltimore). 2019 Jan;98(2):e13906. doi: 10.1097/MD.0000000000013906. PMID: 30633164; PMCID: PMC6336621.
7. Wang M, Shan Y, Wu C, Cao P, Sun W, Han J, Shen L, Chen J, Yu P, Chen X. Efficacy and Safety of Qishen Yiqi Dripping Pill for Heart Failure With Preserved Ejection Fraction: A Systematic Review and Meta-Analysis. Front Pharmacol. 2021 Feb 9;11:626375. doi: 10.3389/fphar.2020.626375. PMID: 33633570; PMCID: PMC7900630.

## **1.5 Embase**

The database search in Embase was carried out on May 03, 2022, and a total of 8 studies were found.

**search strategy**

| No. | Query | Results | Date |
| --- | --- | --- | --- |
| #4 | #1 AND #2 AND #3 | 8 | 03-May-22 |
| #3 | 'meta-analysis' OR 'system assesment' | 357,100 | 03-May-22 |
| #2 | 'qishen yiqi dripping pill' OR 'qishen yiqi' | 68 | 03-May-22 |
| #1 | 'heart failure'/exp OR 'heart failure' OR 'cardiac failure' OR 'heart decompensation' OR 'decompensation, heart' OR 'heart failure, right-sided' OR 'heart failure, right sided' OR 'right-sided heart failure' OR 'right sided heart failure' OR 'myocardial failure' OR 'congestive heart failure' OR 'heart failure, congestive' OR 'heart failure, left-sided' OR 'heart failure, left sided' OR 'left-sided heart failure' OR 'left sided heart failure' | 666,923 | 03-May-22 |


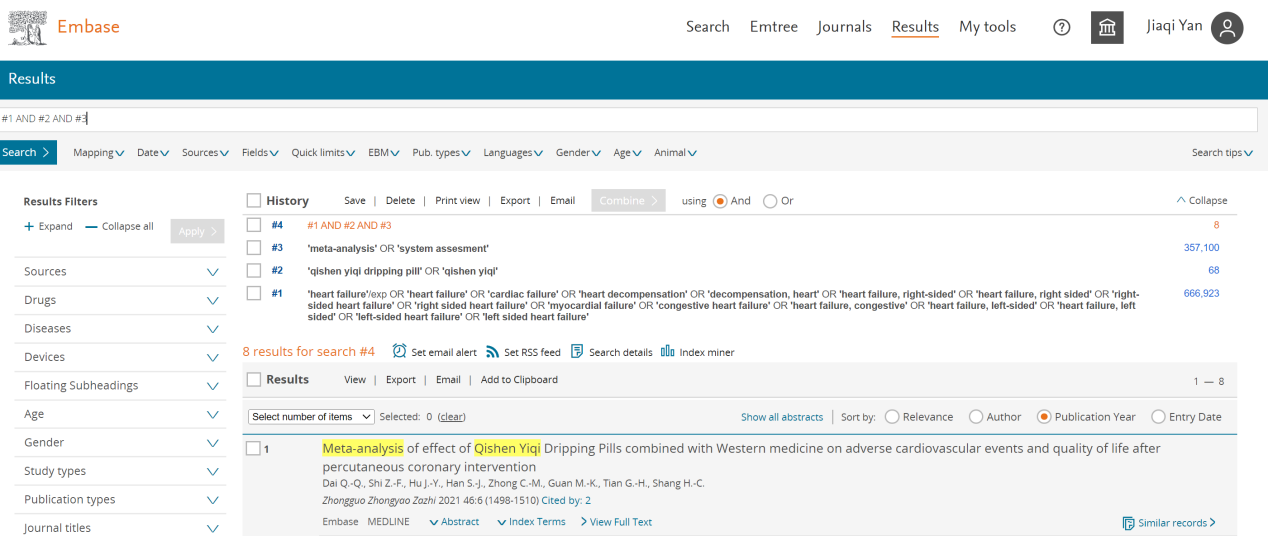


1. Dai QQ, Shi ZF, Hu JY, Han SJ, Zhong CM, Guan MK, Tian GH, Shang HC. [Meta-analysis of effect of Qishen Yiqi Dripping Pills combined with Western medicine on adverse cardiovascular events and quality of life after percutaneous coronary intervention]. Zhongguo Zhong Yao Za Zhi. 2021 Mar;46(6):1498-1510. Chinese. doi: 10.19540/j.cnki.cjcmm.20200618.501. PMID: 33787149.
2. Wang M, Shan Y, Wu C, Cao P, Sun W, Han J, Shen L, Chen J, Yu P, Chen X. Efficacy and Safety of Qishen Yiqi Dripping Pill for Heart Failure With Preserved Ejection Fraction: A Systematic Review and Meta-Analysis. Front Pharmacol. 2021 Feb 9;11:626375. doi: 10.3389/fphar.2020.626375. PMID: 33633570; PMCID: PMC7900630.
3. Guan H, Dai G, Ren L, Gao W, Fu H, Zhao Z, Liu X, Li J. Efficacy and safety of Qishen Yiqi dripping pills as a complementary treatment for Heart Failure: A protocol of updated systematic review and meta-analysis. Medicine (Baltimore). 2021 Jan 15;100(2):e24285. doi: 10.1097/MD.0000000000024285. PMID: 33466215; PMCID: PMC7808537.
4. Chen L, Wang R, Liu H, Wei S, Jing M, Wang M, Zhao Y. Clinical Efficacy and Safety of Qishen Yiqi Dropping Pill Combined with Conventional Western Medicine in the Treatment of Chronic Heart Failure: A Systematic Review and Meta-Analysis. Evid Based Complement Alternat Med. 2021 Feb 2;2021:6612653. doi: 10.1155/2021/6612653. PMID: 33603818; PMCID: PMC7872761.
5. Liu, Junjie PhDa; Xu, Zixuan MSa; Yang, Shuangjie MSb; Du, Kangjia BSb; Zhang, Yili MSb; Tan, Nannan BSb; Sun, Xiaoli PhDc; Zhao, Huihui PhDb,∗; Wang, Wei PhDb,∗ Efficacy and safety of Qishen granules for chronic heart failure, Medicine: December 24, 2020 - Volume 99 - Issue 52 - p e23901 doi: 10.1097/MD.0000000000023901
6. Lin SS, Liu CX, Zhang JH, Wang XL, Mao JY. Efficacy and Safety of Oral Chinese Patent Medicine Combined with Conventional Therapy for Heart Failure: An Overview of Systematic Reviews. Evid Based Complement Alternat Med. 2020 Aug 27;2020:8620186. doi: 10.1155/2020/8620186. PMID: 32908572; PMCID: PMC7474350.
7. Wang H, Li L, Qing X, Zhang S, Li S. Efficacy of Qishen Yiqi Drop Pill for Chronic Heart Failure: An Updated Meta-Analysis of 85 Studies. Cardiovasc Ther. 2020 Sep 22;2020:8138764. doi: 10.1155/2020/8138764. PMID: 33042225; PMCID: PMC7530480.
8. Wang SH, Mao JY, Hou YZ, Wang JY, Wang XL, Li ZJ. [Routine western medicine treatment plus qishen yiqi dripping pill for treating patients with chronic heart failure: a systematic review of randomized control trials]. Zhongguo Zhong Xi Yi Jie He Za Zhi. 2013 Nov;33(11):1468-75. Chinese. PMID: 24483105.

## **1.6 Cochrane Library**

The database search in Cochrane Library was carried out on May 03, 2022, and a total of 0 study was found.

**search strategy**

ID Search

#1 (Heart Failure ):ti,ab,kw OR (Cardiac Failure):ti,ab,kw OR (Heart Decompensation):ti,ab,kw OR (Decompensation, Heart):ti,ab,kw OR (Heart Failure, Right-Sided):ti,ab,kw OR (Heart Failure, Right Sided):ti,ab,kw OR (Right-Sided Heart Failure):ti,ab,kw OR (Right Sided Heart Failure):ti,ab,kw OR (Myocardial Failure):ti,ab,kw OR (Congestive Heart Failure):ti,ab,kw OR (Heart Failure, Congestive):ti,ab,kw OR (Heart Failure, Left-Sided):ti,ab,kw OR (Heart Failure, Left Sided):ti,ab,kw OR (Left-Sided Heart Failure):ti,ab,kw OR (Left Sided Heart Failure):ti,ab,kw(Word variations have been searched)

#2 (qishen yiqi dripping pill):ti,ab,kw OR (qishen yiqi):ti,ab,kw(Word variations have been searched)

#3 (meta-analysis):ti,ab,kw OR (system assesment):ti,ab,kw(Word variations have been searched)

#4 #1 and #2 and #3


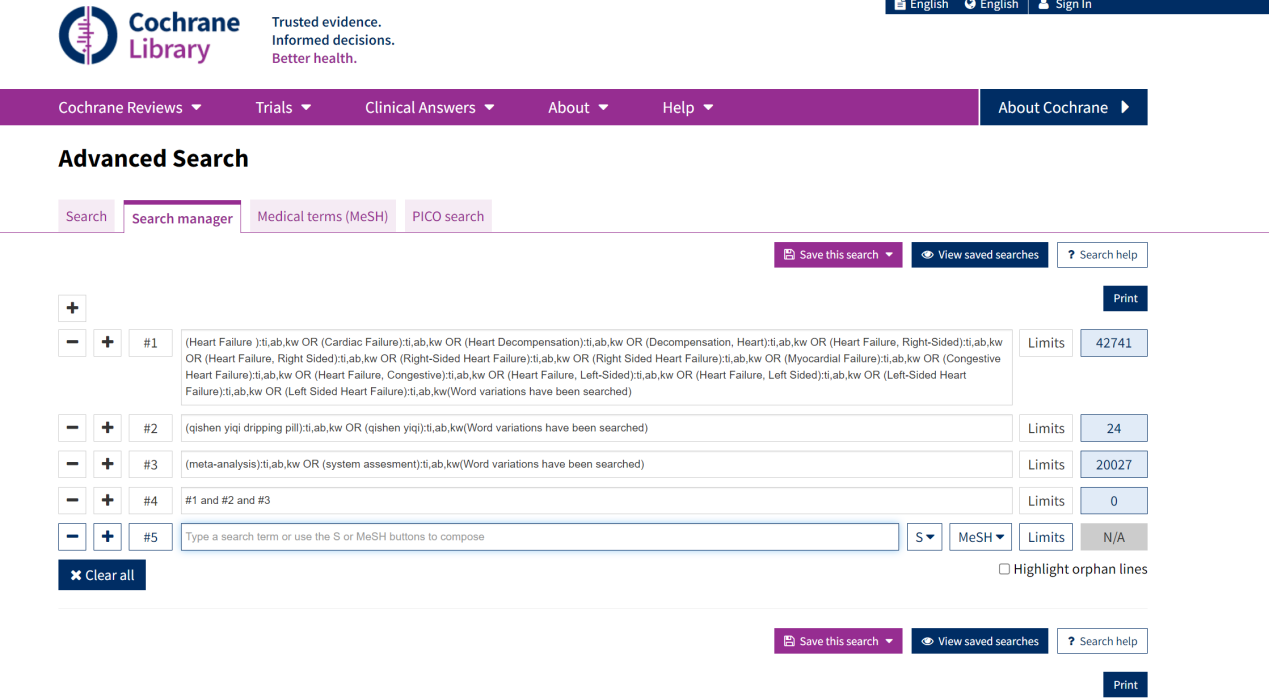


# Supplementary Material S2. Literature screening process

# **List of studies searched after removing duplicate studies**

| 1. Chang M, Cheng L, Shen Y, Zhang Y, Zhang Z, Hao P. Qishenyiqi dripping pill improves ventricular remodeling and function in patients with chronic heart failure: A pooled analysis. Medicine (Baltimore). 2019 Jan;98(2):e13906. doi: 10.1097/MD.0000000000013906. PMID: 30633164; PMCID: PMC6336621. |
| --- |
| 1. Chen Lisheng,Wang Ruilin,Liu Honghong,Wei Shizhang,Jing Manyi,Wang Min,Zhao Yanling,Uddin Md. Sahab. Clinical Efficacy and Safety of Qishen Yiqi Dropping Pill Combined with Conventional Western Medicine in the Treatment of Chronic Heart Failure: A Systematic Review and Meta-Analysis[J]. Evidence-Based Complementary and Alternative Medicine,2021,2021. |
| 1. Guan, Hui et al. “Efficacy and safety of Qishen Yiqi dripping pills as a complementary treatment for Heart Failure: A protocol of updated systematic review and meta-analysis.” Medicine vol. 100,2 (2021): e24285. doi:10.1097/MD.0000000000024285 |
| 1. Lin SS, Liu CX, Zhang JH, Wang XL, Mao JY. Efficacy and Safety of Oral Chinese Patent Medicine Combined with Conventional Therapy for Heart Failure: An Overview of Systematic Reviews. Evid Based Complement Alternat Med. 2020 Aug 27;2020:8620186. doi: 10.1155/2020/8620186. PMID: 32908572; PMCID: PMC7474350. |
| 1. Liu, Junjie PhDa; Xu, Zixuan MSa; Yang, Shuangjie MSb; Du, Kangjia BSb; Zhang, Yili MSb; Tan, Nannan BSb; Sun, Xiaoli PhDc; Zhao, Huihui PhDb,∗; Wang, Wei PhDb,∗ Efficacy and safety of Qishen granules for chronic heart failure, Medicine: December 24, 2020 - Volume 99 - Issue 52 - p e23901 doi: 10.1097/MD.0000000000023901 |
| 1. Wang H, Li L, Qing X, Zhang S, Li S. Efficacy of Qishen Yiqi Drop Pill for Chronic Heart Failure: An Updated Meta-Analysis of 85 Studies. Cardiovasc Ther. 2020 Sep 22;2020:8138764. doi: 10.1155/2020/8138764. PMID: 33042225; PMCID: PMC7530480. |
| 1. Wang M, Shan Y, Wu C, Cao P, Sun W, Han J, Shen L, Chen J, Yu P, Chen X. Efficacy and Safety of Qishen Yiqi Dripping Pill for Heart Failure With Preserved Ejection Fraction: A Systematic Review and Meta-Analysis. Front Pharmacol. 2021 Feb 9;11:626375. doi: 10.3389/fphar.2020.626375. PMID: 33633570; PMCID: PMC7900630. |
| 1. 陈慧,李春,石天娇,等. 芪参益气滴丸对心衰大鼠RAAS系统的实验研究[J]. 北京中医药大学学报,2014,37(8):538-542. DOI:10.3969/j.issn.1006-2157.2014.08.009. |
| 1. 代倩倩,石兆峰,胡嘉元,等. 芪参益气滴丸联合西药治疗对经皮冠状动脉介入术后不良心血管事件及生活质量影响的Meta分析[J]. 中国中药杂志,2021,46(6):1498-1510. DOI:10.19540/j.cnki.cjcmm.20200618.501. |
| 1. 戴小华,王越. 芪参益气滴丸治疗慢性心力衰竭疗效的Meta分析[C]. //第17届中国南方国际心血管病学术会议论文集. 2015:182-183. |
| 1. 单秋月,张闻,吕露,等. 用芪参益气滴丸抑制慢性心力衰竭患者心室重构效果的Meta分析[J]. 当代医药论丛,2017,15(11):71-73. |
| 1. 樊根豪,邢作英,陈召起,张孟孟,胡宇才,赵安社,刘梦琳,王永霞.芪参益气滴丸治疗冠心病心力衰竭的系统评价[J].中国中医基础医学杂志,2020,26(07):932-935+997. |
| 1. 高长春,徐国良,秦玲. 芪参益气滴丸治疗慢性充血性心力衰竭有效性及安全性的Meta分析[J]. 中国中医急症,2014,23(2):232-234. DOI:10.3969/j.issn.1004-745X.2014.02.018. |
| 1. 葛昭,刘春香,林姗姗,等. 芪参益气滴丸治疗射血分数降低心力衰竭疗效及安全性的系统评价[J]. 湖南中医杂志,2022,38(1):115-123. DOI:10.16808/j.cnki.issn1003-7705.2022.01.034. |
| 1. 郭娇,刘新灿,孙天福. 芪参益气滴丸联合西药治疗慢性心力衰竭的临床疗效和安全性分析[J]. 中国循证心血管医学杂志,2020,12(5):519-524. DOI:10.3969/j.issn.1674-4055.2020.05.03. |
| 1. 李雪靖,张慧玲,王晓丽,等. 芪参益气滴丸治疗慢性心力衰竭的循证药物经济学评价[J]. 中国医院用药评价与分析,2020,20(12):1472-1477,1482. DOI:10.14009/j.issn.1672-2124.2020.12.017. |
| 1. 林姗姗. 慢性心力衰竭中医诊疗指南的制定研究[D].天津中医药大学,2021.DOI:10.27368/d.cnki.gtzyy.2021.000338. |
| 1. 刘军刚,顾万红,刘效栓,李喜香,黄清杰.芪参益气滴丸治疗慢性心力衰竭的Meta分析[J].中国新药与临床杂志,2014,33(03):189-195. |
| 1. 牛振超,林文勇,李益萍,等. 芪参益气滴丸辨证施治对慢性心力衰竭疗效影响的Meta分析[J]. 药物评价研究,2021,44(5):1076-1087. DOI:10.7501/j.issn.1674-6376.2021.05.027. |
| 1. 裴英豪,朱翠玲,朱明军,等. 芪参益气滴丸治疗慢性心力衰竭的疗效及安全性系统评价[J]. 中国中医急症,2013,22(9):1472-1475. DOI:10.3969/j.issn.1004-745X.2013.09.007. |
| 1. 曲凤,邢冬梅,郑文科,等. 芪参益气滴丸治疗缺血性心力衰竭的系统评价[J]. 中国实验方剂学杂志,2014,20(3):213-218. DOI:10.11653/syfj2014030213. |
| 1. 田野,顾健霞. 芪参益气滴丸治疗冠心病心力衰竭的Meta分析[J]. 中国中医急症,2016,25(9):1725-1727,1742. DOI:10.3969/j.issn.1004-745X.2016.09.025. |
| 1. 王拴虎,毛静远,侯雅竹,等. 西药常规加用芪参益气滴丸治疗慢性心力衰竭随机对照试验的系统评价[J]. 中国中西医结合杂志,2013,33(11):1468-1475. DOI:10.7661/CJIM.2013.11.1468. |
| 1. 王越,戴小华. 芪参益气滴丸治疗慢性心力衰竭疗效的Meta分析[C]. //中华中医药学会心病分会2015年学术会议论文集. 2015:216-223. |
| 1. 谢锋,段广靖,王斌,等. 芪参益气滴丸联合曲美他嗪治疗慢性心力衰竭的meta分析[J]. 海南医学院学报,2021,27(9):689-694,700. DOI:10.13210/j.cnki.jhmu.20200814.004. |
| 1. 张秀文,戴雁彦,王宗亮,等. 芪参益气滴丸联合曲美他嗪治疗慢性心力衰竭的Meta分析[J]. 世界中西医结合杂志,2021,16(1):23-28,33. DOI:10.13935/j.cnki.sjzx.210105. |
| 1. 章轶立,王娟,李园,等. 芪参益气滴丸治疗慢性心力衰竭(气虚血瘀证)的Meta分析[J]. 中国实验方剂学杂志,2019,25(21):162-169. DOI:10.13422/j.cnki.syfjx.20191447. |
| 1. 朱方敏. 西药常规加用芪参益气滴丸治疗慢性心力衰竭随机对照试验的系统评价分析[J]. 健康之路,2014(8):324-325. DOI:10.3969/j.issn.1671-8801.2014.08.537. |

## **List of studies excluded after reading title and abstract**

**About protocols for meta-analysis**

1. Guan, Hui et al. “Efficacy and safety of Qishen Yiqi dripping pills as a complementary treatment for Heart Failure: A protocol of updated systematic review and meta-analysis.” Medicine vol. 100,2 (2021): e24285. doi:10.1097/MD.0000000000024285
2. Liu, Junjie PhDa; Xu, Zixuan MSa; Yang, Shuangjie MSb; Du, Kangjia BSb; Zhang, Yili MSb; Tan, Nannan BSb; Sun, Xiaoli PhDc; Zhao, Huihui PhDb,∗; Wang, Wei PhDb,∗ Efficacy and safety of Qishen granules for chronic heart failure, Medicine: December 24, 2020 - Volume 99 - Issue 52 - p e23901 doi: 10.1097/MD.0000000000023901

**Participants with non-chronic heart failure**

1. Wang Mengxi,Shan Yiwen,Wu Chenjie,Cao Peihua,Sun Weixin,Han Jie,Shen Le,Chen Jiandong,Yu Peng,Chen Xiaohu. Efficacy and Safety of Qishen Yiqi Dripping Pill for Heart Failure With Preserved Ejection Fraction: A Systematic Review and Meta-Analysis
2. 代倩倩,石兆峰,胡嘉元,等. 芪参益气滴丸联合西药治疗对经皮冠状动脉介入术后不良心血管事件及生活质量影响的Meta分析[J]. 中国中药杂志,2021,46(6):1498-1510. DOI:10.19540/j.cnki.cjcmm.20200618.501.
3. 葛昭,刘春香,林姗姗,等. 芪参益气滴丸治疗射血分数降低心力衰竭疗效及安全性的系统评价[J]. 湖南中医杂志,2022,38(1):115-123. DOI:10.16808/j.cnki.issn1003-7705.2022.01.034.
4. 曲凤,邢冬梅,郑文科,等. 芪参益气滴丸治疗缺血性心力衰竭的系统评价[J]. 中国实验方剂学杂志,2014,20(3):213-218. DOI:10.11653/syfj2014030213.

**Type of study not meta-analysis**

1. 林姗姗. 慢性心力衰竭中医诊疗指南的制定研究[D].天津中医药大学,2021.DOI:10.27368/d.cnki.gtzyy.2021.000338.
2. Chang M, Cheng L, Shen Y, Zhang Y, Zhang Z, Hao P. Qishenyiqi dripping pill improves ventricular remodeling and function in patients with chronic heart failure: A pooled analysis. Medicine (Baltimore). 2019 Jan;98(2):e13906. doi: 10.1097/MD.0000000000013906. PMID: 30633164;
3. 陈慧,李春,石天娇,等. 芪参益气滴丸对心衰大鼠RAAS系统的实验研究[J]. 北京中医药大学学报,2014,37(8):538-542. DOI:10.3969/j.issn.1006-2157.2014.08.009.

**Conference papers**

[1]戴小华,王越. 芪参益气滴丸治疗慢性心力衰竭疗效的Meta分析[C]. //第17届中国南方国际心血管病学术会议论文集. 2015:182-183.

[2]王越,戴小华. 芪参益气滴丸治疗慢性心力衰竭疗效的Meta分析[C]. //中华中医药学会心病分会2015年学术会议论文集. 2015:216-223.

**Interventions are not QSYQ**

1. Lin SS, Liu CX, Zhang JH, Wang XL, Mao JY. Efficacy and Safety of Oral Chinese Patent Medicine Combined with Conventional Therapy for Heart Failure: An Overview of Systematic Reviews. Evid Based Complement Alternat Med. 2020 Aug 27;2020:8620186. doi: 10.1155/2020/8620186.

## **3. List of studies excluded after reading full-text and reasons**

**Type of study not meta-analysis**

朱方敏. 西药常规加用芪参益气滴丸治疗慢性心力衰竭随机对照试验的系统评价分析[J]. 健康之路,2014(8):324-325. DOI:10.3969/j.issn.1671-8801.2014.08.537.

**Not proper comparisons**

章轶立,王娟,李园,等. 芪参益气滴丸治疗慢性心力衰竭(气虚血瘀证)的Meta分析[J]. 中国实验方剂学杂志,2019,25(21):162-169. DOI:10.13422/j.cnki.syfjx.20191447.

## **4. Total studies includes in reviews**

1. 张秀文,戴雁彦,王宗亮,等. 芪参益气滴丸联合曲美他嗪治疗慢性心力衰竭的Meta分析[J]. 世界中西医结合杂志,2021,16(1):23-28,33. DOI:10.13935/j.cnki.sjzx.210105.
2. 谢锋,段广靖,王斌,等. 芪参益气滴丸联合曲美他嗪治疗慢性心力衰竭的meta分析[J]. 海南医学院学报,2021,27(9):689-694,700. DOI:10.13210/j.cnki.jhmu.20200814.004.
3. 王拴虎,毛静远,侯雅竹,等. 西药常规加用芪参益气滴丸治疗慢性心力衰竭随机对照试验的系统评价[J]. 中国中西医结合杂志,2013,33(11):1468-1475. DOI:10.7661/CJIM.2013.11.1468.
4. 田野,顾健霞. 芪参益气滴丸治疗冠心病心力衰竭的Meta分析[J]. 中国中医急症,2016,25(9):1725-1727,1742. DOI:10.3969/j.issn.1004-745X.2016.09.025.
5. 牛振超,林文勇,李益萍,等. 芪参益气滴丸辨证施治对慢性心力衰竭疗效影响的Meta分析[J]. 药物评价研究,2021,44(5):1076-1087. DOI:10.7501/j.issn.1674-6376.2021.05.027.
6. 裴英豪,朱翠玲,朱明军,等. 芪参益气滴丸治疗慢性心力衰竭的疗效及安全性系统评价[J]. 中国中医急症,2013,22(9):1472-1475. DOI:10.3969/j.issn.1004-745X.2013.09.007.
7. 郭娇,刘新灿,孙天福. 芪参益气滴丸联合西药治疗慢性心力衰竭的临床疗效和安全性分析[J]. 中国循证心血管医学杂志,2020,12(5):519-524. DOI:10.3969/j.issn.1674-4055.2020.05.03.
8. 高长春,徐国良,秦玲. 芪参益气滴丸治疗慢性充血性心力衰竭有效性及安全性的Meta分析[J]. 中国中医急症,2014,23(2):232-234. DOI:10.3969/j.issn.1004-745X.2014.02.018.
9. Chen Lisheng,Wang Ruilin,Liu Honghong,Wei Shizhang,Jing Manyi,Wang Min,Zhao Yanling,Uddin Md. Sahab. Clinical Efficacy and Safety of Qishen Yiqi Dropping Pill Combined with Conventional Western Medicine in the Treatment of Chronic Heart Failure: A Systematic Review and Meta-Analysis[J]. Evidence-Based Complementary and Alternative Medicine,2021,2021.
10. Wang H, Li L, Qing X, Zhang S, Li S. Efficacy of Qishen Yiqi Drop Pill for Chronic Heart Failure: An Updated Meta-Analysis of 85 Studies. Cardiovasc Ther. 2020 Sep 22;2020:8138764. doi: 10.1155/2020/8138764. PMID: 33042225; PMCID: PMC7530480.
11. 樊根豪,邢作英,陈召起,张孟孟,胡宇才,赵安社,刘梦琳,王永霞.芪参益气滴丸治疗冠心病心力衰竭的系统评价[J].中国中医基础医学杂志,2020,26(07):932-935+997.
12. 单秋月,张闻,吕露,等. 用芪参益气滴丸抑制慢性心力衰竭患者心室重构效果的Meta分析[J]. 当代医药论丛,2017,15(11):71-73.
13. 李雪靖,张慧玲,王晓丽,等. 芪参益气滴丸治疗慢性心力衰竭的循证药物经济学评价[J]. 中国医院用药评价与分析,2020,20(12):1472-1477,1482. DOI:10.14009/j.issn.1672-2124.2020.12.017.
14. 刘军刚,顾万红,刘效栓,李喜香,黄清杰.芪参益气滴丸治疗慢性心力衰竭的Meta分析[J].中国新药与临床杂志,2014,33(03):189-195.

## Supplementary Material S3.**Details of risk of bias assessment.**

| study ID | Chan qiuyue 2017 | Chen Lisheng 2021 | Fan Genhao 2020 | Gao Changchun 2014 | Guo Jiao 2020 | Li Xuejing 2020 | Liu Jungang 2014 | Niu Zhenchao 2021 | Pei Yinghao 2013 | Tian Ye 2016 | Wang Hao 2020 | Wang Shuanhu 2013 | Xie Feng 2021 | Zhang Xiuwen 2021 |
| --- | --- | --- | --- | --- | --- | --- | --- | --- | --- | --- | --- | --- | --- | --- |
| Phase 2: Identifying concerns with the review process |  |  |  |  |  |  |  |  |  |  |  |  |  |  |
| Signalling question |  |  |  |  |  |  |  |  |  |  |  |  |  |  |
| 1.1 Did the review adhere to pre-defined objectives and eligibility criteria? | PY | PY | PY | PY | PY | PY | PY | PY | PY | PY | N | PY | PY | PY |
| 1.2 Were the eligibility criteria appropriate for the review question? | Y | Y | Y | Y | Y | Y | Y | Y | Y | Y | N | Y | Y | Y |
| 1.3 Were eligibility criteria unambiguous? | Y | Y | Y | Y | Y | Y | Y | Y | Y | Y | N | Y | Y | Y |
| 1.4 Were all restrictions in eligibility criteria based on study characteristics appropriate? | Y | Y | Y | Y | Y | Y | Y | Y | Y | Y | N | Y | Y | Y |
| 1.5 Were any restrictions in eligibility criteria based on sources of information appropriate? | Y | Y | Y | Y | Y | Y | Y | Y | Y | Y | N | Y | Y | Y |
| Concerns regarding specification of study eligibility criteria | L | L | L | L | L | L | L | L | L | L | H | L | L | L |
| **Signalling question** |  |  |  |  |  |  |  |  |  |  |  |  |  |  |
| 2.1 Did the search include an appropriate range of databases/ electronic sources for published and unpublished reports? | N | N | N | N | N | N | N | N | N | N | N | N | N | N |
| 2.2 Were methods additional to database searching used to identify relevant reports? | N | N | N | Y | Y | N | N | N | N | N | N | Y | N | N |
| 2.3 Were the terms and structure of the search strategy likely to retrieve as many eligible studies as possible? | N | N | N | N | N | N | N | N | N | N | N | N | N | N |
| 2.4 Were restrictions based on date, publication format, or language appropriate? | PY | PY | PY | PY | PY | PY | PY | PY | PY | PY | PY | PY | PY | PY |
| 2.5 Were efforts made to minimize errors in selection of studies? | Y | Y | Y | Y | N | Y | Y | Y | Y | Y | Y | Y | Y | Y |
| Concerns regarding methods used to identify and/or select studies | H | H | H | H | H | H | H | H | H | H | H | H | H | H |
| **Signalling question** |  |  |  |  |  |  |  |  |  |  |  |  |  |  |
| 3.1 Were efforts made to minimize error in data collection? | NI | Y | NI | NI | NI | NI | NI | Y | Y | Y | Y | Y | NI | Y |
| 3.2 Were sufficient study characteristics available for both review authors and readers to be able to interpret the results? | N | Y | Y | Y | Y | Y | Y | Y | Y | Y | Y | Y | Y | Y |
| 3.3 Were all relevant study results collected for use in the synthesis? | Y | Y | Y | Y | Y | Y | Y | Y | Y | Y | Y | Y | Y | Y |
| 3.4 Was risk of bias (or methodological quality) formally assessed using appropriate criteria? | Y | Y | Y | Y | Y | Y | Y | Y | Y | Y | Y | Y | Y | Y |
| 3.5 Were efforts made to minimize error in risk of bias assessment? | Y | Y | N | N | Y | Y | Y | Y | Y | Y | Y | Y | Y | Y |
| Concerns regarding methods used to collect data and appraise studies | H | L | H | H | U | U | U | L | L | L | L | L | U | L |
| **Signalling question** |  |  |  |  |  |  |  |  |  |  |  |  |  |  |
| 4.1 Did the synthesis include all studies that it should? | Y | Y | Y | Y | Y | N | N | Y | N | Y | Y | N | N | Y |
| 4.2 Were all predefined analyses followed or departures explained? | NI | NI | NI | NI | NI | NI | NI | NI | NI | NI | NI | NI | NI | NI |
| 4.3 Was the synthesis appropriate given the nature and similarity in the research questions, study designs and outcomes across included studies? | Y | Y | Y | Y | Y | Y | Y | Y | Y | Y | Y | Y | Y | Y |
| 4.4 Was between-studies variation (heterogeneity) minimal or addressed in the synthesis? | N | Y | Y | Y | N | N | N | Y | N | Y | Y | Y | Y | Y |
| 4.5 Was robustness of the finding(s) assessed e.g. through funnel plot or sensitivity analyses? | N | N | N | N | N | Y | N | Y | N | Y | Y | N | N | N |
| 4.6 Were biases in primary studies minimal or addressed in the synthesis? | N | Y | N | Y | Y | N | N | Y | N | Y | Y | Y | Y | Y |
| Concerns regarding the synthesis and findings | H | H | H | H | H | H | H | U | H | U | U | H | H | H |
| **Phase 3: Judging risk of bias** |  |  |  |  |  |  |  |  |  |  |  |  |  |  |
| **Domain** |  |  |  |  |  |  |  |  |  |  |  |  |  |  |
| 1. Concerns regarding specification of study eligibility criteria | L | L | L | L | L | L | L | L | L | L | H | L | L | L |
| 2. Concerns regarding methods used to identify and/or select studies | H | H | H | H | H | H | H | H | H | H | H | H | H | H |
| 3. Concerns regarding used to collect data and appraise studies | H | L | H | H | U | U | U | L | L | L | L | L | U | Y |
| 4. Concerns regarding the synthesis | H | H | H | H | H | H | H | U | H | U | U | H | H | H |
| **RISK OF BIAS IN THE REVIEW** |  |  |  |  |  |  |  |  |  |  |  |  |  |  |
| **Signalling question** |  |  |  |  |  |  |  |  |  |  |  |  |  |  |
| A. Did the interpretation of findings address all of the concerns identified the Phase 2 assessment? | N | N | N | N | N | N | N | N | N | N | N | N | N | N |
| B. Was the relevance of identified studies to the review's research question appropriately considered? | Y | Y | Y | Y | Y | Y | Y | Y | Y | Y | Y | Y | Y | Y |
| C. Did the reviewers avoid emphasizing results on the basis of their statistical significance? | Y | Y | Y | Y | Y | Y | Y | Y | Y | Y | Y | Y | Y | Y |
| Risk of bias in the review | H | H | H | H | H | H | H | H | H | H | H | H | H | H |
